# The network interplay of interferon and Toll-like receptor signaling pathways in the anti-*Candida* immune response

**DOI:** 10.1038/s41598-021-99838-0

**Published:** 2021-10-13

**Authors:** Ranieri Coelho Salgado, Dennyson Leandro M. Fonseca, Alexandre H. C. Marques, Sarah Maria da Silva Napoleao, Tábata Takahashi França, Karen Tiemi Akashi, Caroline Aliane de Souza Prado, Gabriela Crispim Baiocchi, Desirée Rodrigues Plaça, Gabriel Jansen-Marques, Igor Salerno Filgueiras, Roberta De Vito, Paula Paccielli Freire, Gustavo Cabral de Miranda, Niels Olsen Saraiva Camara, Vera Lúcia Garcia Calich, Hans D. Ochs, Lena F. Schimke, Igor Jurisica, Antonio Condino-Neto, Otavio Cabral-Marques

**Affiliations:** 1 Department of Immunology, Institute of Biomedical Sciences, University of São Paulo, São Paulo, Brazil; 2 Department of Clinical and Toxicological Analyses, School of Pharmaceutical Sciences, University of São Paulo, São Paulo, SP Brazil; 3 Information Systems, School of Arts, Sciences and Humanities, University of Sao Paulo, São Paulo, Brazil; 4 Department of Biostatistics and the Data Science Initiative at, Brown University, Providence, RI USA; 5 Department of Pediatrics, University of Washington School of Medicine, and Seattle Children’s Research Institute, Seattle, WA USA; 6 Osteoarthritis Research Program, Division of Orthopedic Surgery, Schroeder Arthritis Institute, UHN; Data Science Discovery Centre, Krembil Research Institute, UHN; Departments of Medical Biophysics and Computer Science, University of Toronto, Toronto, Canada; 7 Institute of Neuroimmunology, Slovak Academy of Sciences, Bratislava, Slovakia; 8 Network of Immunity in Infection, Malignancy, and Autoimmunity (NIIMA), Universal Scientific Education and Research Network (USERN), São Paulo, SP Brazil

**Keywords:** Immunology, Diseases, Medical research

## Abstract

Fungal infections represent a major global health problem affecting over a billion people that kills more than 1.5 million annually. In this study, we employed an integrative approach to reveal the landscape of the human immune responses to *Candida* spp*.* through meta-analysis of microarray, bulk, and single-cell RNA sequencing (scRNA-seq) data for the blood transcriptome. We identified across these different studies a consistent interconnected network interplay of signaling molecules involved in both Toll-like receptor (TLR) and interferon (IFN) signaling cascades that is activated in response to different *Candida* species (*C. albicans*, *C. auris*, *C. glabrata*, *C. parapsilosis*, and *C. tropicalis*). Among these molecules are several types I IFN, indicating an overlap with antiviral immune responses. scRNA-seq data confirmed that genes commonly identified by the three transcriptomic methods show cell type-specific expression patterns in various innate and adaptive immune cells. These findings shed new light on the anti-*Candida* immune response, providing putative molecular pathways for therapeutic intervention.

## Introduction

Fungal infections, including the emergence of new fungal pathogens highly resistant to antifungal drugs, represent a major global health issue^1–5^. Indeed, fungal infections affect over a billion individuals worldwide and kill more than 1.5 million annually. Among these diseases, invasive candidiasis (IC) is the most common, affecting approximately 250,000 people annually and causing more than 50,000 deaths^6,7^. Overall, an increasing number of patients with malignancies, inborn errors of immunity (IEIs), autoimmune diseases (involving immunosuppressive treatment), and hematopoietic stem cell or organ transplantation is contributing to the high frequency of individuals susceptible to life-threatening fungal pathogens^8,9^. Thus, a better understanding of molecular pathways that can be explored to develop new therapies to reduce the morbidity and mortality caused by *Candida* infections is needed^10,11^.

Linear and mechanistic approaches have elegantly demonstrated that the antifungal immune response involves appropriate recognition of pathogen-associated molecular patterns (PAMPs) by different pattern recognition receptors (PRRs) expressed on the host cell membrane, such as C-type lectin receptors (CLRs: dectin-1, dectin-2, and CD209), scavenger receptors (CD36), and Toll-like receptors (TLRs), e.g., TLR2 and TLR4. Additionally, intracellular PRRs, including RIG-I-like receptors (RLRs: melanoma differentiation-associated protein 5 or MDA5), TLRs (e.g., TLR3 and TLR9), and NOD-like receptors (NLRs: nucleotide-binding oligomerization domain-containing protein or NOD1/2, NOD-, LRR- and pyrin domain-containing 3 or NLRP3), are relevant and expressed by antigen-presenting cells and phagocytes, which bind well-known ligands^12–14^. Activation of PRRs induces several signaling events, e.g., the canonical nuclear factor (NF)-κB pathway^15^, that trigger effector antifungal mechanisms. Such mechanisms include phagocytosis, reactive oxygen species (ROS) production^16^, degranulation, and neutrophil extracellular traps (NETs)^17,18^. Moreover, PRRs promote production of key inflammatory cytokines, such as tumor necrosis factor (TNF)-α, interleukin (IL)-1β, IL-6, IL-17, type I interferons (IFNs [IFN-α/β]), and the IL-12/IFN-γ axis^11,14,19,20^, shaping and guiding immune cells^14^.

Nevertheless, the landscape of antifungal molecules remains to be resolved in an integrative manner. To reach this goal, we performed multistudy analysis of blood transcriptome microarray, bulk, and single-cell RNA sequencing (scRNA-seq) data to uncover the landscape of human immune responses to *Candida* spp. Our approach provides new insight into the anti-*Candida* immune response.

## Results

### Multilayered conservation of TLR and IFN signaling pathways in response to *C. albicans*

**Figure 1:**
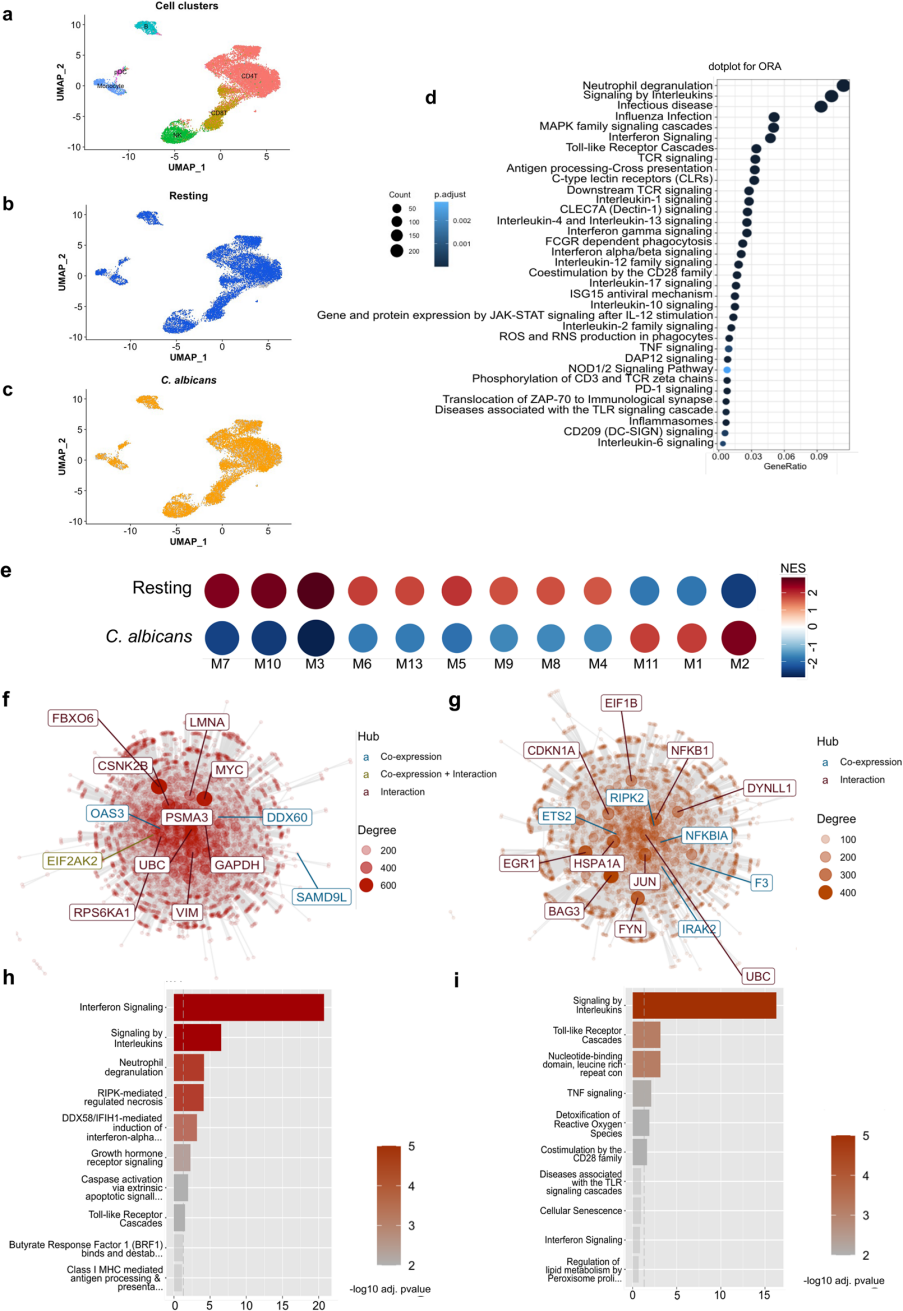
Multilayered induction of TLR and IFN signaling pathways in response to *C. albicans*. (**a**), UMAP visualization of scRNAseq profiles colored according to cell cluster. (**b**) and (**c**)**,** UMAP of resting and *C. albicans-*activated cell groups. DEGs, differentially expressed genes; IFN, interferon; ORA, overrepresentation analysis; scRNAseq, single-cell RNA sequencing; TLR, Toll-like receptor; UMAP, uniform manifold approximation and projection. (**d**), Dot plot showing pathways associated with the immune response to *C. albicans*, as obtained by ORA of DEGs. (**e**), Coexpression modules significantly enriched (M1-M11, and M13) in PBMCs (resting n = 30; *C. albicans* infected n = 24; dataset GSE42606)**.** (**f**) and (**g**) Network representation of M1 and M2 with hubs (most connected genes) colored based on coexpression (blue color), coexpression and interactions (green color), or interactions only (dark-red color). (**h**) and (i)**,** Enrichment representation obtained by modular genes coexpression in M1 and M2 showing significantly (− Log10 transformed adjusted *p* value) enriched signaling pathways. *IFN, interferon; TLR, Toll-like receptor.*

We surveyed published RNA-seq datasets and found 8 related to the human immune response to *Candida* spp., including 5 microarray and 2 bulk RNA-seq datasets and one scRNA-seq dataset (further details are provided in the Methods section). First, we explored scRNA-seq by performing overrepresentation analysis (ORA) of differentially expressed genes (DEGs) from innate immune (monocytes, natural killer, and plasmacytoid dendritic cells) and adaptive (CD4 + , CD8 + , and CD19 + lymphocytes) cells. The DEGs were assigned to clusters as previously described^21^ (Fig. 1a) under resting and *C. albicans* conditions (Fig. 1b–c), and 6722 DEGs (Suppl. Table S1) were identified. Enriched pathways associated with the immune response to *C. albicans* are shown in Fig. 1d; all enriched categories are present in Suppl. Table S2. Among them were 72 and 99 DEGs belonging to TLR and IFN (both type I and type II) signaling cascades, respectively. Among the DEGs, 62 are involved in both TLR and IFN signaling cascades based on our enrichment analysis or as previously reported in the literature (Suppl. Table S3).

We next sought to determine whether interplay between TLR and IFN signaling cascades is induced at a topological level. To this end, we performed modular gene coexpression analysis^22^ using the microarray dataset from Smeekens et al^11^, a unique public dataset containing more than 15 samples per group (30 resting and 24 *C. albicans*-activated samples) to obtain biologically meaningful modular networks^23^. Modular gene coexpression analysis using CEMiTool^24^ identified thirteen enriched coexpression modules from all genes expressed by PBMCs (which contain lymphocyte subpopulations, monocytes, and dendritic cells). Among these modules, 12 were significantly enriched (9 downregulated and 3 upregulated) in response to *C. albicans* infection (Fig. 1e)*.* Of note, modules M1 and M2 indicate gene coexpression and upregulation of IFN and interleukin signaling with TLR cascades (Fig. 1f–i).

Based on the results obtained by modular coexpression analysis, we dissected the significantly enriched pathways of differentially expressed genes (DEGs) induced by *C. albicans*^11^. In agreement with the topological results obtained using CEMiTool, ORA of DEGs using the ClusterProfiler tool^25^ pinpointed different clusters related to activation of TLR and IFN signaling (Suppl. Fig. 1a–b). The relationship between the 30 most enriched pathways and their associated genes is shown in a network view in Suppl. Fig. 1c, and the entire list of all enriched pathways is summarized in Suppl. Table S4. Type I IFN signaling was the most significant pathway modulated by *C. albicans*, as previously reported by Smeekens et al.^11^ and as recently characterized by Bruno et al.^26^. Furthermore, stimulation by *C. albicans* resulted in significant enrichment of several TLR signaling events, such as TLR4, TLR3, TLR7/8, and TLR9, MyD88/TIR-domain-containing adapter-inducing interferon-β (TRIF)/TIR domain containing adaptor protein (TIRAP) cascades, as well as TRAF6-mediated NF-κB activation. ORA also indicated that *C. albicans* activates chemokine (G protein-coupled receptor [GPCR] ligand binding) and cytokine (IL-10, IL-3 and IL4) signaling pathways, IFN-α/β signaling, the interferon-stimulated gene 15 (ISG15) antiviral mechanism, TNF receptor-associated factor 3 (TRAF3)-dependent IRF activation, DExD/H-box helicase 58 (DDX58)/interferon-induced with helicase C domain 1 (IFIH1)-mediated induction of IFN-α/β, and regulation of type I and II IFN (Suppl. Fig. 1a and c; Suppl. Table S4). This finding agrees with the study performed by Jaeger et al.^27^, who characterized in detail the relevance of IFIH1 (MDA5) in the anti-*Candida* immune response.

### *C. albicans* infection activates common TLR- and IFN-associated genes in peripheral blood leukocytes

**Figure 2:**
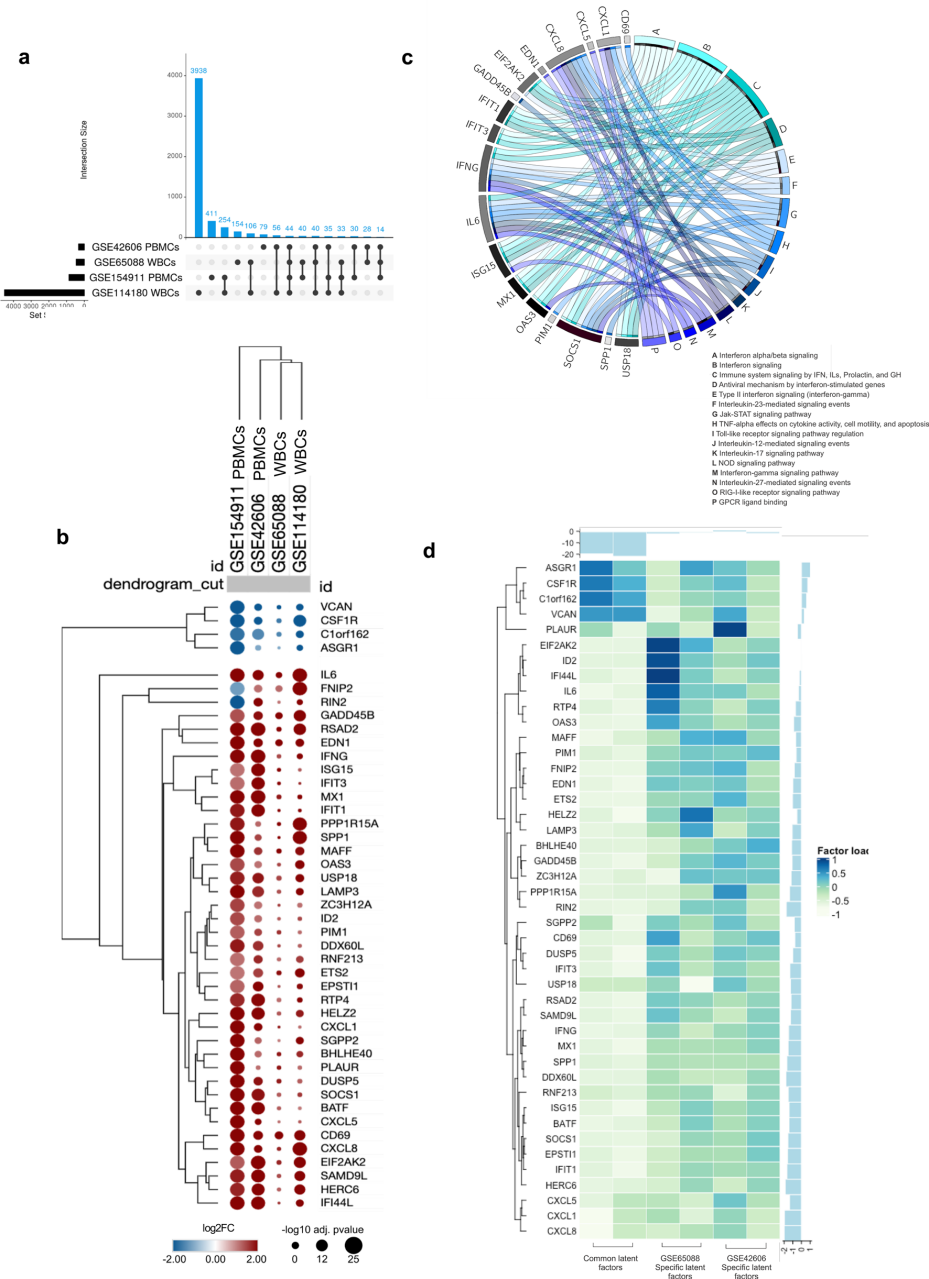
*C. albicans* activates common TLR- and IFN-associated genes in peripheral blood leukocytes. (**a**) The upper plot displays the number (set size) of DEGs present in each dataset (y-axis: WBCs, GSE65088, and GSE114180; PBMCs: GSE42606 and GSE154911) and their intersections. Black bubbles, present in rows, mark the dataset, which refers to the amount present in the blue columns, with intersections between two or more groups being shown. (**b**), Hierarchical clustering of the 44 common DEGs demonstrating gene expression patterns across different studies. The size and color of circles correspond to − Log10 transformed adjusted *p* value and Log2-fold change (Log2FC), respectively. Blue represents downregulated DEGs, and red indicates upregulated DEGs. The cutoff applied to identify the down/upregulated genes was Log2FC <  − 1/ > 1 and adjusted *p* value < 0.05. Rows and columns were clustered based on cosine similarity between Log2FC values. (**c**), GOplot of selected immunological pathways and associated genes. (**d**), Heatmap of common and specific latent factors across studies. Heatmaps contain genes presenting positive and negative loadings ranging from − 1 to 1. *DEGs*, *differentially expressed genes; PBMCs, peripheral blood mononuclear cells; WBCs, white blood cells.*

We further investigated which DEGs and signaling pathways are consistently activated by *C. albicans* in peripheral blood leukocytes such as PBMCs (Smeekens et al.^11^ and Bruno et al.^28^) and peripheral whole blood cells (WBCs, Dix et al.^29^ and Sieber & Kämmer et al.^30,31^) in all publicly available datasets*.* WBCs contain PBMCs (lymphocytes 20–45% and monocytes 2–10%) and granulocytes (neutrophils: 50–70%; basophils: 0–1%; and eosinophils: 1–5%)^32^. Meta-analysis of WBC and PBMC gene expression datasets using the P value combination method revealed 44 commonly activated DEGs (40 upregulated and 4 downregulated) (Fig. 2a, Suppl. Table S5). These DEGs form well-defined hierarchical clusters according to cell populations, i.e., PBMC datasets presented a closer expression pattern among them and WBC datasets when comparing both regulation and significance (Fig. 2b). Enrichment analyses using EnrichR of these 44 genes revealed 87 significantly affected pathways (Suppl. Table S6), including TLR and IFN-α/β signaling (Fig. 2c). Furthermore, these 44 DEGs were enriched in other interleukin signaling pathways, such as JAK-STAT, IL-12, IL-17, IL-23, TNF, chemokines (GPCR ligand binding) and PRRs, including RIG-I-like receptor and NOD signaling. Multistudy factor analysis of eligible^33^ datasets (WBCs: Dix et al.^29^ and PBMCs: Smeekens et al.^11^; those with the minimal number of samples required for this analysis) identified two common latent factors with high loadings, whereas specific latent factors showed low loadings across these studies, strengthening the biological relevance of the 44 common genes (Fig. 2d, Suppl. Table S7).

### *C. albicans* activates common TLR and IFN signaling pathways across different layers of immunity

**Figure 3:**
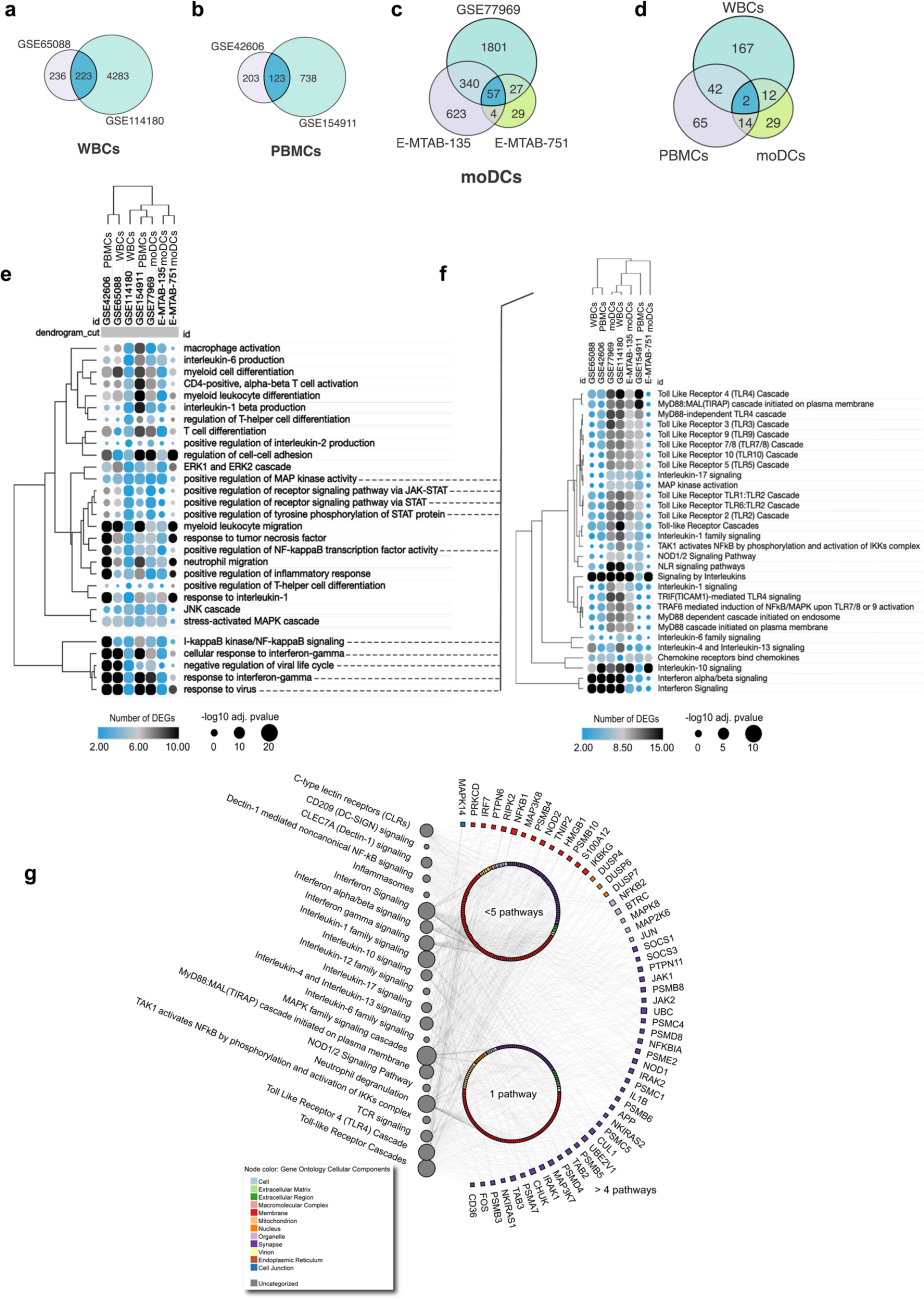
*C. albicans* activate common TLR and IFN signaling pathways across different leukocyte populations. (**a**–**c**), Proportional Venn diagrams displaying the number of DEGs present in each dataset grouped by cell type and their intersections: datasets of WBCs (**a**), PBMCs (**b**), and moDCs (**c**). (**d**), The intersection plot highlights the number of common DEGs across different cell groups (Venn diagrams were created using CorelDraw2019, available at coreldraw.com). (**e**), Hierarchical clustering exhibiting pathways enriched in common biological processes across studies (Suppl. Table S10). (**f**) Further analysis of TLR- and IFN-associated pathways. In both heatmaps, the size of the circles corresponds to the adjusted p value transformed into -Log10, and the color intensity indicates the number of genes in each biological process and pathway across studies. (**g**) Network demonstrating interactions between TLR- and IFN-associated DEGs/signaling pathways with other molecules and signaling cascades classically associated with antifungal immune responses. Enrichment analysis was performed using Reactome. Circular nodes represent pathways, and their size denotes the number of genes enriching the pathways. Colored squares represent the cellular location of genes. Genes interacting with more than 5 pathways are named. The interaction network was built using NAViGaTOR software. DEGs, differentially expressed genes; moDCs, monocyte-derived dendritic cells; IFN, interferon; PBMCs, peripheral blood mononuclear cells; TLR, Toll-like receptor; WBCs, whole Blood Cells.

We further included monocyte-derived dendritic cell (moDC) studies (Dix et al., Rizzetto et al., and Rizzetto et al.)^34–36^ in our integrative analysis. moDCs are known to be essential players in antifungal immunity, bridging the innate and adaptive arms of the immune system. We searched for genes commonly regulated by *C. albicans* in the transcriptomes of WBCs, PBMCs, and moDCs under resting and *C. albicans* activation conditions. Intersection analyses performed according to cell population identified 123, 223, and 57 common DEGs for the WBC, PBMC, and moDC datasets, respectively (Fig. 3a–c). Five genes (RIN2, RGL1, MARCKS, FNIP2, and TLR7) reported as commonly differentially expressed in PBMC studies were upregulated in the dataset of Smeekens et al.^11^ and downregulated in that of Bruno et al.^28^. All other common DEGs were consistently up- or downregulated across studies investigating the same cell population (Suppl. Table S9).

However, only 2 common DEGs were present across all seven datasets (Fig. 3d), which by themselves did not show significant signaling pathway enrichment. We then explored whether the DEGs from each dataset are enriched in common signaling biological processes among all studies. Gene Ontology (GO) analysis using ClusterProfiler revealed 173 common biological processes (Suppl. Table S8), and we found several molecules/pathways to be essential for the antifungal immune response^12^, including a cluster of IFN-γ and NF-kB signaling and a previously described overlap with the immune response to viruses^11^ (Fig. 3e). The latter has been mechanistically characterized by Bourgeois et al.^37^. Additional ORA of DEGs involved in this cluster showed significant enrichment in signaling cascades of single TLRs (TLR2, TLR3, TLR4, TLR5, TLR9, TLR9, and TLR10), TLR heterodimers (TLR1/TLR2, TLR2/TLR6, TLR7/8), and TLR adapter molecules (MyD88/TIRAP, TRAF6, TRIF) as well as in several interleukin signaling pathways, such as IL-1, IL-4/IL-13, IL-6, IL-10, IL-17, and IFN-α/β (Fig. 3f). Furthermore, 1096 DEGs (Suppl. Table S11) affecting common biological processes among WBCs, PBMCs and moDCs were detected. In Fig. 3g, the interactome obtained from some of these 1096 DEGs and enriched signaling cascades is illustrated (Suppl. Table S12), highlighting the association of TLR- and IFN-signaling cascades consistently enriched in our analyses. These 1096 DEGs were also enriched in other PRR and interleukin signaling pathways, including CLRs (dectin-1), NLRs (NOD1/2), pro- (IL-1, IL6, IL-17, IL-12), anti-inflammatory (IL-10), and T helper 2 (IL-4 and IL-13) cytokines. This immunological balance between pro- and anti-inflammatory events is crucial for properly controlling fungal infections while maintaining immune homeostasis^38,39^.

### *C. albicans* infection increases correlation between TLR- and IFN-associated genes

**Figure 4:**
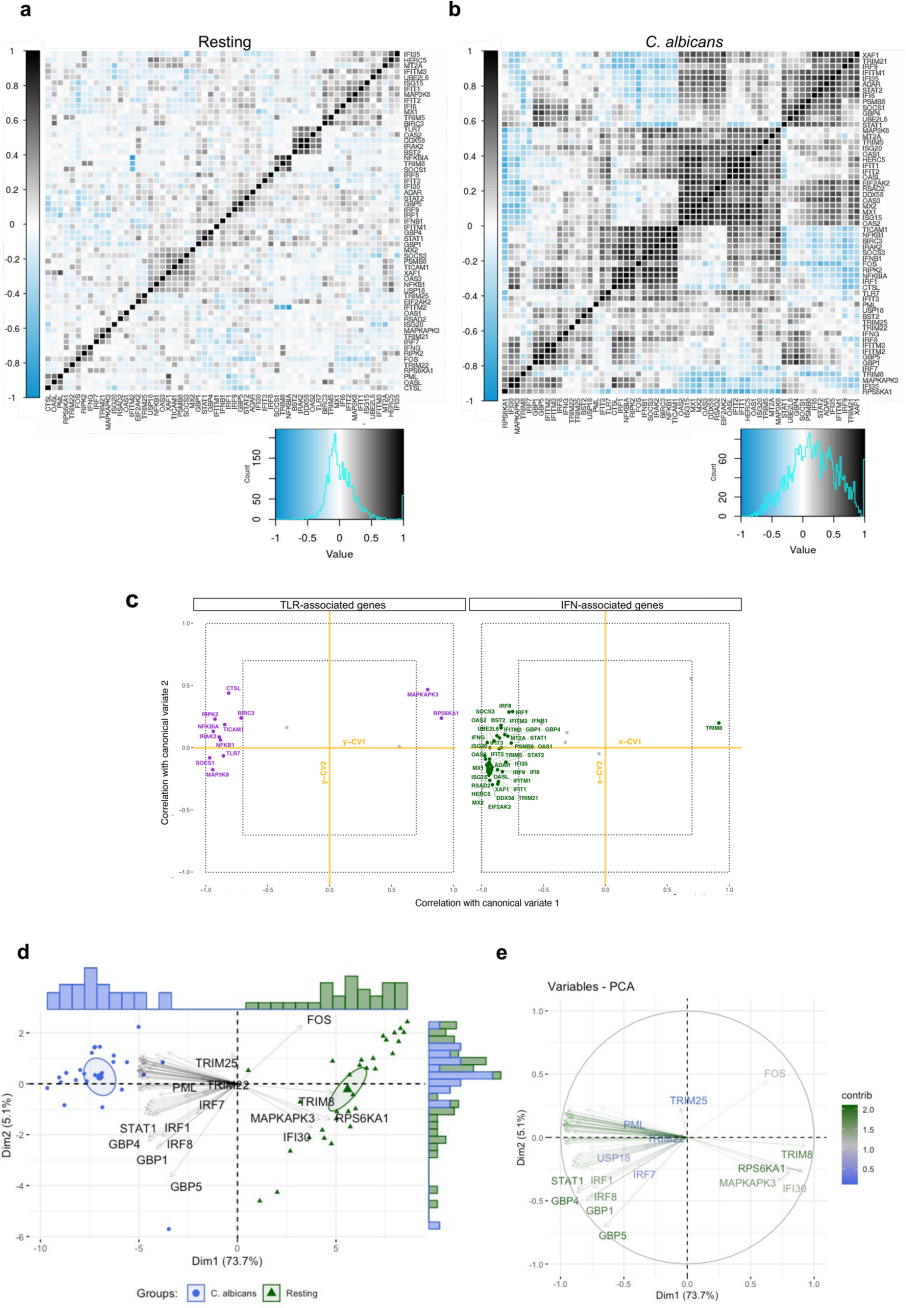
Relationship between molecules associated with TLR and IFN signaling cascades. (**a**) and (**b**), Correloplot of DEGs associated with TLR and IFN signaling cascades in PBMCs (GSE42606) in the (**a**)**,** absence or (**b**)**,** presence of *C. albicans.* Histograms of Pearson’s correlation coefficient, containing negative and positive correlations from 1 to − 1, respectively. (**c**) Estimated correlations of TLR- and IFN-associated DEGs versus their corresponding first 2 canonical variates (x-CV1 and x-CV2 for IFN-associated genes; y-CV1 and y-CV2 for TLR-associated genes). Gray-colored variables (with names omitted) are those with correlation coefficients ≤ 0.7 in two corresponding canonical variates. Inner dotted lines limit the canonical correlation coefficient between − 0.7 and 0.7; outer dotted lines limit the coefficient between − 1 and 1**.** (**d**) and (**e**), PCA was used for stratification analysis of resting and *C. albicans*-infected PBMCs based on TLR- and IFN-associated DEGs. (**d**), Of note, individuals with similar expression values for these DEGs are grouped together; (**e**) Variables with positive correlation are pointing to the same side of the plot; negatively correlated variables point to opposite sides. *DEGs, differentially expressed genes; IFN, interferon; PBMCs, peripheral blood mononuclear cells; TLR, Toll-like receptor.*

After verifying TLR and type I and II IFN signaling cascade consistency, we assessed the degree of association between these two variables in the immune response to *C. albicans*. Due to the requirement of minimum sample size^40^, we selected TLR- and IFN-associated genes present in the PBMC transcriptome data from Smeekens et al.^11^. This dataset contains 45 and 14 TLR- and IFN-associated DEGs modulated by *C. albicans* compared to the resting group. Overall, *C. albicans* infection increased mainly positive correlations between TLR- and IFN-associated DEGs (Fig. 4a–b). We then performed canonical correlation analysis (CCA), a generic parametric model used to quantify relationships between two groups of interrelated and interdependent variables^41^, to further assess the association strength between TLR and IFN DEGs. This approach revealed a pair of canonical variates (x-CV1 and y-CV1), a finding that highlights the strong association between most TLR- and IFN-associated DEGs in both resting and *C. albicans*-infected PBMCs (Fig. 4c) as well as the ability to stratify these conditions (Fig. 4d–e).

### Interplay between TLR and IFN signaling pathways is conserved in response to nonalbicans *Candida* species

**Figure 5:**
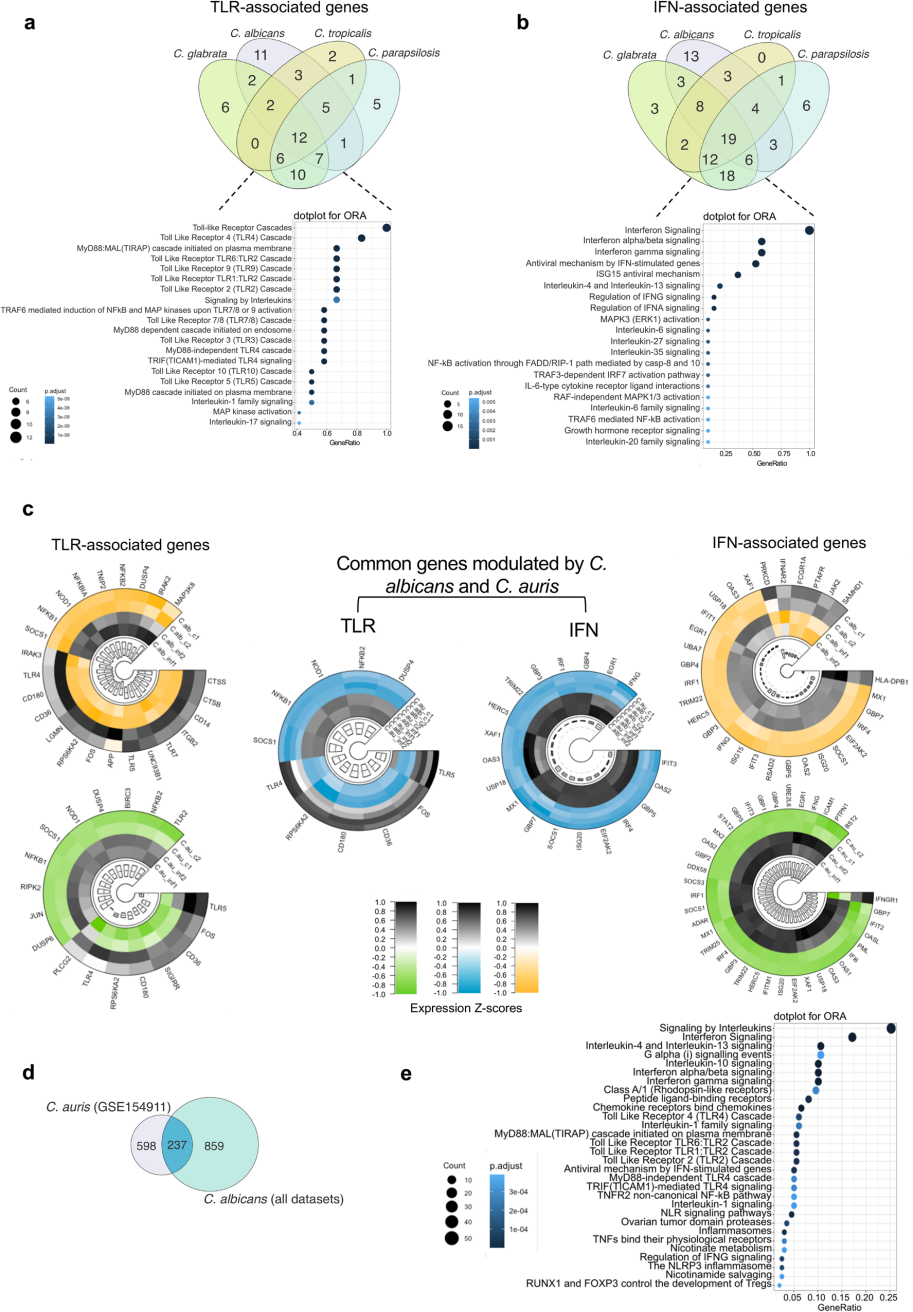
Induction of interplay between TLR and IFN signaling pathways by other *Candida* species. Venn diagram showing the transcriptional overlap between (**a**), TLR- and (**b**), IFN-associated DEGs and the signaling pathways enriched in response to nonalbicans *Candida* species (*C. glabrata*, *C. parapsilosis*, and *C. tropicalis*) in comparison to *C. albicans* (created using CorelDraw2019, available at coreldraw.com). (**c**), Circular heatmaps of RNAseq expression z-scores computed for log2 transformed DEGs (*p* value adj < 0.05, fold change > 1 and <  − 1) compare expression of TLR (left panels) and IFN (right panels) signaling pathways induced by *C. albicans* (green/gray heatmaps) or *C. auris* (yellow/gray heatmaps) all from GSE154911. Small circular heatmaps (blue/gray) demonstrate common DEGs modulated by *C. abicans* and *C auris*. Individual circular heatmaps were created using the R packages circlize and ComplexHeatmap; the figure layout was edited using CorelDraw2019. (**d**), Venn diagram showing the transcriptional overlap (an intersection containing 237 shared DEGs) induced by *C. auris* and *C. albicans* (those 1096 genes found across all studies: Suppl. Table S11). (**e**), Dotplot of enriched signaling pathways by the 237 shared DEGs. *DEGs, differentially expressed genes; IFN, interferon; ORA, overrepresentation analysis; TLR, Toll-like receptor.*

We then assessed whether only *C. albicans* induces the observed correlation between TLR- and IFN-associated genes or other nonalbicans *Candida* species, such as *C. glabrata*, *C. parapsilosis*, *C. tropicalis*, and multidrug-resistant *C. auris*^28,42^. We used the public dataset of Sieber & Kämmer et al.^30,31^, and intersection analyses performed with TLR- and IFN-associated DEGs from this dataset revealed 12 and 19 common DEGs, respectively, upon activation by the different species. These DEGs are involved in several pathways related to both TLR and IFN signaling (Fig. 5a–b, Suppl. Table S13).

We determined whether this interplay between the TLR and IFN signaling pathways is involved in the response to multidrug-resistant *C. auris* by exploring a unique publicly available dataset analyzing the immune response to *C. auris* and *C. albicans* (Bruno et al.^28^). Similar to activation by *C. albicans*, ORA of DEGs induced by *C. auris* included TLR and IFN cascades among the 30 most enriched pathways (Suppl. Fig. 2a–b). *C. albicans* and *C. auris* similarly modulated levels of DEGs involved in TLR signaling, including NF-κB1, NF-κB2, JUN, and DUSP4, and in IFN signaling, such as IRFs, GBPs, SOCS1, ISG20, TRIM, and IFIT3 (Fig. 5c). When we compared the DEGs induced by *C. auris* with enriched common pathways among all datasets (1096 DEGs, Suppl. Table S11) assessing the immune response to *C. albicans,* we identified 237 common DEGs (Fig. 5d), and ORA of these common DEGs indicated that the interplay between TLR and IFN signaling cascades is a consistent immunologic feature in response to these two species of *Candida* (Fig. 5e).

### Inborn errors of immunity (IEIs) corroborate interplay between TLR and IFN signaling cascades

Finally, we aimed to evaluate the potential clinical and translational relevance of the identified TLR- and IFN-associated genes and molecular pathways consistently modulated by *Candida*. Therefore, we searched for IEI-associated genes known to increase susceptibility to both systemic and mucocutaneous candidiasis in humans, as described by Tangye et al.^43^. For this analysis, we only included mutations but not single-nucleotide polymorphisms (SNPs) associated with susceptibility to *Candida* infection. Jaeger et al.^27^ found that MDA5 SNPs are associated with *Candida*, and mutations in this gene have been reported as an IEI associated with increased susceptibility to recurrent viral infections^43^. Regardless, this fact reinforces type I interferon’s consistent role in the anti-*Candida* immune response, indicating that patients with increased susceptibility to *Candida* spp. need to be screened for mutations in MDA5.

**Figure 6:**
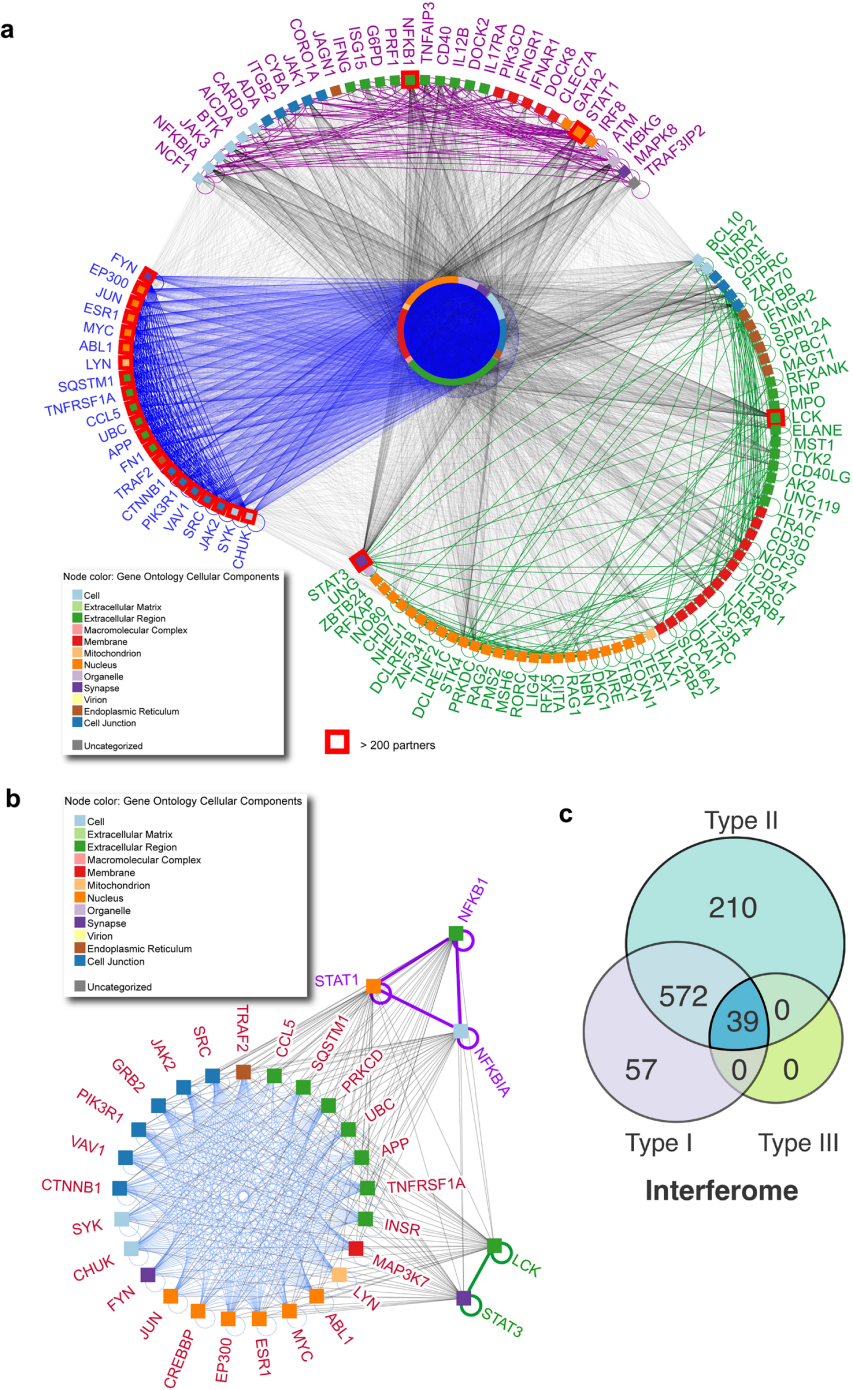
The interactome of DEGs enriched in signaling pathways involved in the anti-*Candida* immune response and its association with inborn errors of immunity. (**a**), Relationships (edges) among the 1096 DEGs (nodes) found across all studies (Suppl. Table S11). Subnetworks (semicircles) represent genes associated with IEI causing increased susceptibility to candidiasis; 34 purple node genes are shared with the group of 1096 DEGs, and 66 green nodes represent those not found in the *Candida* datasets. Blue nodes are highlighted genes with more than 200 partners of interaction. Colored squares and circles represent the cell location of genes. The interaction network was built using NAViGaTOR software. (**b**), Network of hubs present in (**a**). (**c**), Proportional Venn diagram (created using CorelDraw2019, available at coreldraw.com) of interferon types associated with the group of 1096 DEGs. Interferome analysis revealed 878 IFN-regulated genes modulated by IFN types I, II, and III, as shown in the Venn diagram. *DEGs,* *differentially expressed genes; IFN, Interferon; IEIs, inborn errors of immunity; TLR, Toll-like receptor.*

To date, mutations in 100 genes known to be associated with IEIs have been identified as enhancing susceptibility to candidiasis and other clinical manifestations (Suppl. Table S16). We compared them with the 1096 genes described above (Suppl. Table S11, i.e., those enriching the common biological processes activated by *C. albicans* (Fig. 3e), which encode molecules present in different compartments, such as extracellular regions, organelles, and nuclei, forming macromolecular complexes. Together, these factors form a highly interconnected physical protein–protein interaction network (Fig. 6a) that contains several hubs^44^ (Fig. 6b) defined as having more than or equal to 200 interaction partners. Of note, 34 genes associated with IEIs were also present across the studies. Although 66 genes associated with IEI were not identified in the datasets, these genes were highly connected with the other DEGs in this network. Furthermore, the 1096 DEGs mostly comprised type I and II IFN-associated genes, 878 in total (Fig. 6c, Suppl. Table S14).

The 34 IEI-associated genes present in this network consist of 7 groups of IEIs, including congenital defects of phagocytes, defects of intrinsic and innate immunity, predominantly antibody deficiencies, and diseases of immune dysregulation, as defined by International Union of Immunological Societies Expert Committee (IUIS)^43^ (Suppl. Fig. 3a). Notably, among the hubs are *STAT1*, *STAT3*, *NFKBIA* (*IκBa*), and NFKB1, which are well known to be associated with TLR and IFN signaling^45–50^. ORA of these 34 genes indicated that in addition to dectin-1^51^ and NLR signaling, they were mostly enriched in components of both type I and II IFN and several TLR (TLR1/2, TLR2/6, MyD88, and TRAF6-mediated NF-κB activation) signaling pathways (Suppl. Fig. 3b–c).

### Common TLR- and IFN-associated DEGs and signaling pathways across microarray, bulk, and single-cell RNA-seq datasets

**Figure 7:**
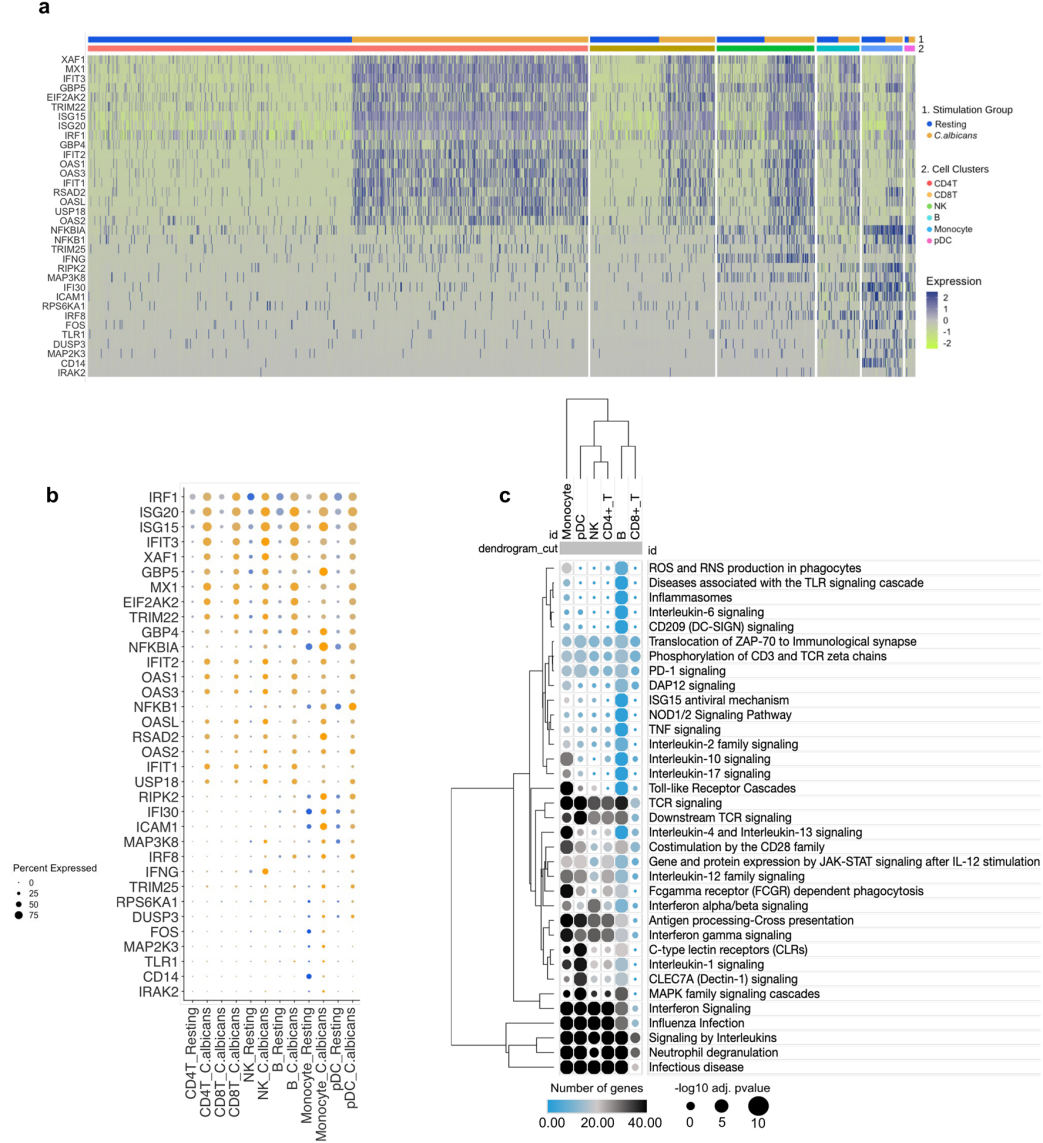
Common TLR- and IFN-associated DEGs and signaling pathways across microarray, bulk, and single-cell RNA-seq datasets. (**a**), Heatmap using expression values from scRNAseq of DEGs also present in microarray and bulk studies; the cell condition and group are indicated by different colors. (**b**), Hierarchical clustering of average expression comparing resting and *C. albicans*-activated cells. (**c**), Hierarchical clustering showing common pathways selected from Fig. 1d across the cell groups; the size of circles corresponds to adjusted p value transformed into − Log10, and the color intensity indicates the number of genes in each pathway across the cell groups. *DEGs, differentially expressed genes; IFN, interferon; TLR, Toll-like receptor; scRNA-seq, single-cell RNA sequencing.*

**Figure 8:**
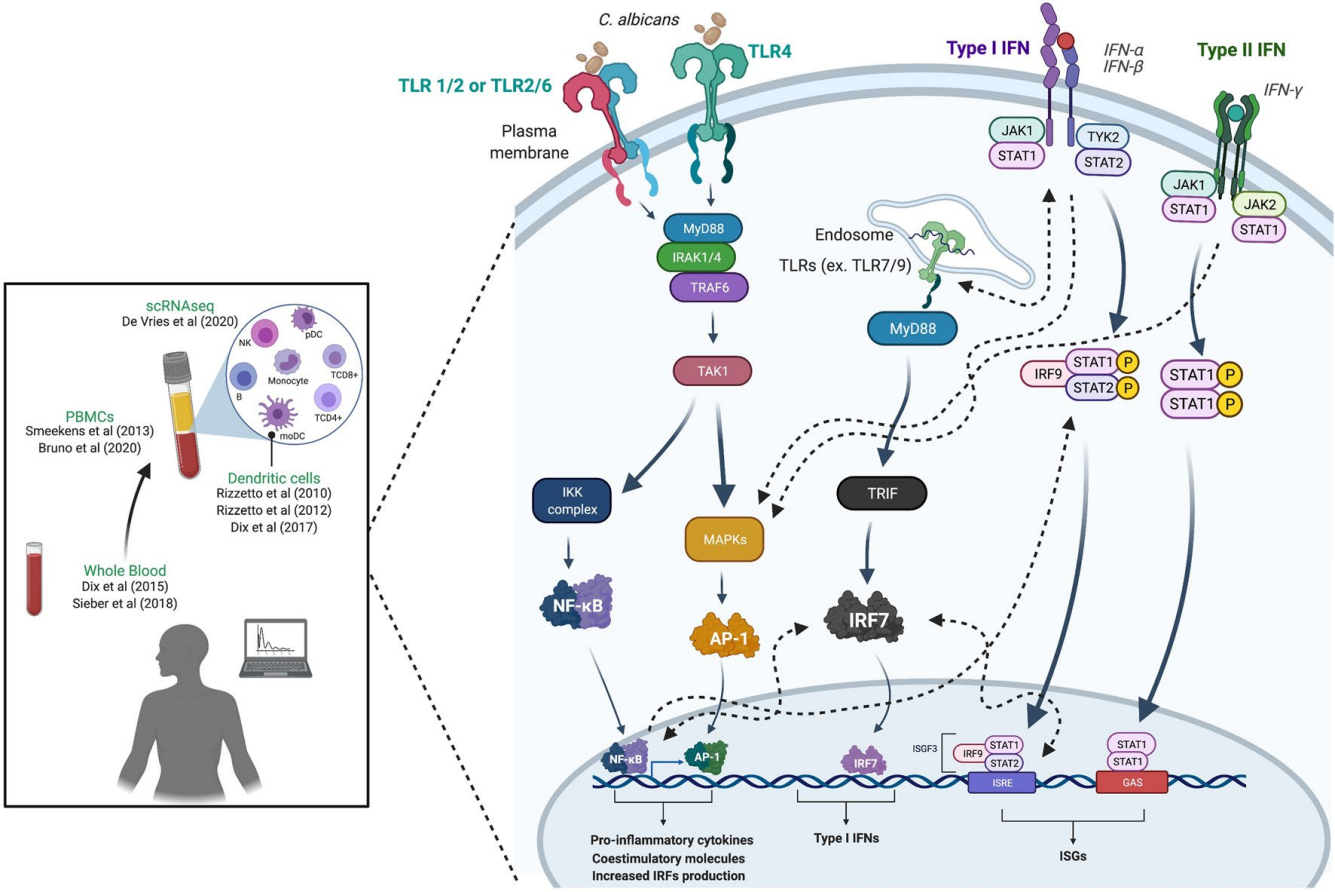
Schematic view summarizing studies and the interplay between TLR and IFN signaling pathways in the immune response to *C. albicans.* The pathways shown are reported in the literature^50^ (created using BioRender.com). *IFN, interferon; TLR, Toll-like receptor.*

Finally, we revisited the scRNA-seq data and found 11 TLR- and 23 IFN-associated DEGs to be among the WBC, PBMC and moDC DEGs identified by microarray and bulk RNA-seq datasets (Suppl. Table S15). Thus, the network interplay of TLR- and IFN-associated DEGs is not a particular feature of a specific leukocyte cell population, as *C. albicans* systemically activated this network in different innate (monocytes, natural killer, and plasmacytoid dendritic cells) and adaptive (CD4 + , CD8 + , and CD19 + lymphocytes) cells identified by the scRNA-seq dataset. Fig. 7a–b illustrates these 34 common genes across the leukocyte subpopulations and those present in the WBC, PBMC, and moDC datasets (Fig. 2a–c). In general, hierarchical clustering of common enriched pathways across the cell subpopulations identified by scRNA-seq revealed a similar upregulation pattern of TLR- and IFN-associated signaling pathways, forming clusters (Fig. 7c), as indicated by microarray and bulk RNA-seq data. Taken together, these data strongly support the immunobiological relevance of interplay between TLR and IFN signaling cascades, as previously described^50^ (Fig. 8).

## Discussion

The association between PRR activation and cytokine production by immune cells is crucial for an adequate immune response to pathogens, and this association has been well investigated by linear approaches or strategies designed to identify the antifungal transcriptomic signature^11,28,52,53^. For instance, several immunologic molecules and pathways, such as those triggered by TLR and IFN, which induce the generation of T cell subpopulations (e.g., T helper 1 [Th1], Th17, and T regulatory [Treg] cells), have been successfully characterized by individual studies and mechanistic approaches^14^. Our systems immunology approach integrates various transcriptomic studies investigating the anti-*Candida* immune response*,* highlighting the consistency of crosstalk between PRRs (e.g., CLRs, TLRs, and NLRs) and type I and type II interferons as well as the cytokine (e.g., TNF and IL-10) cascades previously reported^37,54–60^. In addition, we show consistent overlap between antiviral and antifungal immune responses, supporting the previously reported pivotal role of IFN type I in the immune response to *C. albicans*^11^*.*

Of note, recent reports suggest that *Candida*-specific induction of type I IFN responses is not limited to professional immune cells, as in vivo studies confirm the role of type I IFN in epithelial host defense against *C. albicans*^61,62^. In agreement, a transcriptional profiling study showed that the type I IFN response is induced by vaginal epithelial cells in response to a variety of *Candida* species^63^. Although the type I IFN response has a protective role at early stages of infection, opposite effects (tissue damage) are observed at later stages. This is in line with several studies showing that IFNs may play beneficial or detrimental roles in the host defense against candidiasis. That is, cytokines are essential for controlling the development of severe *Candida* infections by promoting the inflammatory action of phagocytic cells (monocytes and neutrophils), but uncontrolled activation of these cells by IFNs during sepsis may contribute to fatal tissue damage^64–67^. Therefore, understanding the TLR-IFN network at different timepoints of *Candida* infection will provide valuable insight for the clinical management of patients.

It has been suggested that different TLRs synergistically activate immune cells to, for instance, induce expression of several proinflammatory molecules through cooperation of the NF-κB, IRF, STAT, MAPK, ITAM, and PI3K signaling pathways^68–70^. On the one hand, TLR-induced NF-κB signaling promotes the production of several key cytokines, including IFNs, that activate the STAT1-mediated signaling pathway^71^. On the other hand, IFN-γ promotes expression of genes encoding TLRs^72–75^. IFNs also potentiate TLR-induced gene transcription by creating a primed chromatin environment via histone acetylation that allows sustained occupancy by the transcription factors STAT1 and IRF-1 at promotors and enhancers at *TNF*, *IL-6,* and *IL12B* loci^53^. Our phenomenological study confirms these previously reported mechanistic studies and provides new insight into the molecular network of TLR and IFN signaling pathways in the anti-*Candida* immune response. These networks need to be further investigated in other mycoses (paracoccidioidomycosis, histoplasmosis, and cryptococcosis) and neglected diseases (dengue, Zika, leishmaniasis, and Chagas disease) occurring in developing countries^76^. The TLR and IFN interactomes involve more complex events than previously thought, demanding further bottom-up and top-down system immunology investigations.

Our conclusions are based on the integration of publicly available human transcriptomes that identified common DEGs, biological processes and signaling pathways consistently modulated across several leukocyte subpopulations in response to fungal pathogens (*Candida* spp.). Among these DEGs, we highlight those involved in IFN-α/β (e.g., ISGs, IRFs, SOCS, and GBPs), TLR3,4,7/8,9, and TRAF-mediated NF-κB signaling cascades, and the correlation of DEGs involved in these signature clusters increase upon stimulation with *C. albicans*. Of note, among the consistently identified DEGs are those previously associated with IEI that increase host susceptibility to fungal infections, such as those causing chronic mucocutaneous candidiasis. Furthermore, these DEGs are involved in immunological pathways related to the development of IEI phenocopies, such as those targeted by anti-IL-17 or anti-IL17RA autoantibodies, enhancing susceptibility to *Candida* spp. infections. Because the outcome of fungal infections depends primarily on the host immune response, it is highly relevant to examine IEIs that predispose patients toward *Candida* infections^14,52^. IEIs represent an essential research field that has been most useful for investigating human susceptibility models to infection, often revealing the nonredundant role of genes involved in immunologic homeostasis^77–79^. Of the 416 molecular defined IEIs recently summarized by the expert committee of the IUIS, more than 20 syndromes were recognized as being associated with susceptibility to fungal infections^43^. This list of genes associated with an increased risk of fungal infections includes genes regulating signaling via the IL-2 receptor, NF-kB activation, IFN-induced signaling, STAT activation, and TLR signaling. These observations support the relevance of the interactome and interplay events characterized by our analysis, increasing the understanding of consistent immunologic pathways essential for the immune response to *Candida* infections.

However, our manuscript has some limitations that need to be considered, including the different *C. albicans* strains used in the studies, the timepoint of in vitro stimulation, the multiplicity of infection (MOI) applied, or whether heat-killed/inactivated or live organisms were used. These factors may have affect the host immune response in different ways^80,81^, such as in the case of dataset GSE154911^28^ for which a different *C. albicans* strain (CWZ10061110) that only induces a robust transcriptional change after 24 h of stimulation was used. It will be important that future studies address the impact of these factors on transcriptional dynamics in response to *Candida* pathogens. Nevertheless, the fact that we found activation of a network between interferon and Toll-like receptor signaling across several datasets indicates that these interactions reflect an important crosstalk mechanism during the anti-*Candida* immune response. Furthermore, only a few datasets related to *Candida* spp. are available compared with the large amount of data investigating other pathogens, which reinforces the need for more studies related to fungal infections.

Altogether, our work provides a systems immunology view of the interactome of antifungal molecules, revealing a consistent network interplay between TLR and IFN signaling pathways in response to *Candida* spp. This study also indicates new biomarkers and provides novel insight into the systemic immunological mechanism against fungal infections. Future investigations dissecting this interplay will pave the way for new immunotherapy approaches to reduce the high mortality caused by fungal infections. Finally, our study indicates that exploration of functional genomic approaches by applying systems immunology methods to investigate IEI will provide new opportunities for further understanding the immune system *in natura*.

## Methods

### Datasets and curation

We performed an integrative analysis by searching the NCBI GEO database^82^ and ArrayExpress database^83^ to identify publicly available gene expression data of infection by *C. albicans*, *C. auris*, *C. glabrata*, *C. parapsilosis*, and *C. tropicalis* using WBCs, PBMCs, and moDCs. This search comprised studies published between March 2010 and July 2020. Since transcriptome datasets from patients with candidiasis were not publicly available, we applied the following criteria for inclusion: (1) gene expression data for WBCs, PBMCs, and moDCs of in vitro infection with *C. albicans* or *Candida* nonalbicans species; (2) studies composed of at least 2 samples per group; and (3) inclusion of control groups for comparison. All gene expression analysis platforms were considered, and only studies that provided transcriptome data were included for the integrative analyses. Exclusion criteria were (1) nonhuman samples, (2) treatment before molecular genetic analysis, and (3) review studies. RNAseq and MicroArray studies were included in our integrative analysis; five studies were retrieved from the NCBI GEO database^82^ (GSE65088^29^ and GSE114180^30^, GSE42606^11^, GSE154911^28^, and GSE77969^34^) and two from the ArrayExpress database^83^ (E-MTAB-135^35^, E-MTAB-751^36^). Additionally, a single-cell RNA-seq study was included^84^. Information about sample identification and information is provided in Suppl. Tables S17 and S18.

### Single-cell RNASeq analysis

We obtained the Seurat object containing scRNA-seq data from De Vries et al.^84^, which was deposited in the single-cell eQTLGen Consortium database (https://eqtlgen.org/candida.html). We followed the default Seurat pipeline (https://satijalab.org/seurat/articles/pbmc3k_tutorial.html) as previously described by Stuart et al.^85^ to perform differential expression analysis and data visualization (UMAP, dotplot, and heatmap).

### Differential expression analysis of bulk RNA-seq and microarray data

To characterize the immunological signature from global transcriptional profiles in infection by *C. albicans*, read counts of each RNA-seq study were transformed (log2 count per million), and the NetworkAnalyst 3.0 webtool (https://www.networkanalyst.ca/)86 was used to perform differential expression analysis, applying the DESeq2 pipeline. The microarray studies were analyzed through the GEO2R web application^87^, which is available at http://www.ncbi.nlm.nih.gov/geo/geo2r/, using the limma-voom pipeline^88^. We used the statistical cutoffs of log2-fold-change > 1 (upregulated) or < −1 (downregulated) and adjusted p value < 0.05 to select up- and downregulated genes between the *C. albicans* infection and normal groups.

### Analysis of gene co-expression modules

We utilized the GSE42606 dataset to analyze the gene coexpression modules with the R package CEMiTool 1.12.2 using default parameters^24^.

### Enrichment analysis and data visualization

We used the differentially expressed genes (DEGs) to identify enriched GO terms. Pathways and biological processes were identified through overrepresentation analysis (ORA) and EnrichR^89^, and significantly enriched immunological terms were generated according to an adjusted *p* value < 0.05. UpSet and Venn graphs demonstrating intersections and comparisons between common DEGs among the datasets were generated through the webtools Intervene^90^ and Bioinformatics & Evolutionary Genomics (http://bioinformatics.psb.ugent.be/webtools/Venn/). We plotted the set of genes shared between the dataset in bubble-based heat maps, applying one minus cosine similarity through the webtool Morpheus (https://software.broadinstitute.org/morpheus/)91. We used ClusterProfiler^25^ to obtain dot plots of enriched terms associated with *Candida* spp. ClusterProfiler and ORA were performed in R software version 4.0.2 (https://www.r-project.org/index.html) through the packages DOSE, enrichplot, reactomePA, and clusterprofiler^25^. GOplot was plotted using the R packages unikn, circlize, and GOplot^92^. Statistical graphs were constructed using the functionalities of the ggplot2 package^93^. We represent shared DEGs between different fungal infections (*C. albicans* and *C. auris*) with circular heatmaps using the R packages circlize and ComplexHeatmap^94^.

### Correlation analysis

We used the GSE42606 dataset to perform correlation analysis between genes associated with TLRs and type I and II IFN signaling cascades. Correlation matrices were generated with the webtool Intervene^90^ (https://intervene.readthedocs.io/en/latest/index.html) using the Pearson coefficient. Canonical correlation analysis (CCA)^95^ was applied to investigate patterns of association between IFN and TLR genes from the same dataset. CCA was performed on R software version 4.0.2 (https://www.r-project.org/index.html) through the packages CCA and whitening^95^. Principal component analysis (PCA) was carried out using the R functions prcomp and princomp with the factoextra package.

### Molecular network

Networks of pathways related to fungal infection immune responses and physical protein–protein interaction (PPI) networks of DEGs found across all datasets were annotated, analyzed and visualized using NAViGaTOR 3.0,14^96^. Node color represents the Gene Ontology cellular component, as indicated in figure legends. DEGs were used as input for Integrated Interactions Database (IID version 2020–05; http://ophid.utoronto.ca/iid)97,98 to identify direct physical protein interactions. Networks were exported in SVG file format and finalized in Adobe Illustrator 2021.

### Multistudy factor analysis (MSFA)

MSFA is a generalized version of factor analysis that allows for joint analysis of multiple studies. MSFA estimates shared factors common to all studies, as well as factors specific to individual studies. Estimation of parameters for the MSFA model can be computed using either a frequentist or a Bayesian approach. Compared with frequentist analysis, the Bayesian approach offers two major advantages: (1) it provides better-defined factors, and (2) it chooses the dimension of the common and study-specific factors through a practical and useful approach. We adopted a Bayesian multistudy^99^ for inferential analysis to identify common and study-specific factors^14,33,100^ shared by GSE65088 and GSE42606. The Bayesian MSFA considers all data at once in an integrated approach, estimating parameters by maximum-likelihood analysis^101^.

### Interferome analysis

Identification of interferome genes was performed with Interferome V2.01 (http://www.interferome.org/interferome/home.jspx).

### Single-cell RNA-seq differential expression analysis

The Seurat package was used to obtain DEGs between different cell types under infection by *C. albicans* and resting conditions. Enrichment of DEGs by cell group and by total DEGs was performed according to the ClusterProfiler package.

## Supplementary Information

Supplementary Information 1.

Supplementary Information 2.

## Data Availability

The published transcriptome datasets can be found in the GEO and Array Express databases (IDs. GSE65088, GSE114180, GSE42606, GSE154911, GSE77969, E-MTAB-135, E-MTAB-751). Single-cell data are available as Seurat Object on doi 10.1371/journal.ppat.1008408.

## Code availability

R codes used in this work are available at https://github.com/ranieri131/SalgadoRC_CANDIDA_IMMUNE_RESPONSE_2021

## References

[1] Chow NA (2018). Multiple introductions and subsequent transmission of multidrug-resistant Candida auris in the USA: A molecular epidemiological survey. Lancet Infect. Dis..

[2] World Health Organization. GLASS report: early implementation 2017–2018. https://www.who.int/publications/i/item/9789241515061 (2019).

[3] Casadevall A (2017). Don’t forget the fungi when considering global catastrophic biorisks. Heal. Secur..

[4] Warnock DW (2006). Fungal diseases: An evolving public health challenge. Med. Mycol..

[5] Meis JF, Chowdhary A (2018). Candida auris: A global fungal public health threat. Lancet Infect. Dis..

[6] Zeng Z (2019). Surveillance study of the prevalence, species distribution, antifungal susceptibility, risk factors and mortality of invasive candidiasis in a tertiary teaching hospital in Southwest China. BMC Infect. Dis..

[7] Kullberg BJ, Arendrup MC (2015). Invasive candidiasis. N. Engl. J. Med..

[8] Lee, P. P. & Lau, Y.-L. Cellular and molecular defects underlying invasive fungal infections—revelations from endemic mycoses. *Front. Immunol.* **8**, (2017). 10.3389/fimmu.2017.00735 PMC5487386 28702025

[9] Cheng S-C, Joosten LAB, Kullberg B-J, Netea MG (2012). Interplay between Candida albicans and the mammalian innate host defense. Infect. Immun..

[10] Johnson MD (2012). Cytokine gene polymorphisms and the outcome of invasive candidiasis: A prospective cohort study. Clin. Infect. Dis..

[11] Smeekens SP (2013). Functional genomics identifies type I interferon pathway as central for host defense against Candida albicans. Nat. Commun..

[12] Romani L (2011). Immunity to fungal infections. Nat. Rev. Immunol..

[13] Patin EC, Thompson A, Orr SJ (2019). Pattern recognition receptors in fungal immunity. Semin. Cell Dev. Biol..

[14] Netea MG, Joosten LAB, van der Meer JWM, Kullberg B-J, van de Veerdonk FL (2015). Immune defense against Candida fungal infections. Nat. Rev. Immunol..

[15] Liu T, Zhang L, Joo D, Sun S-C (2017). NF-κB signaling in inflammation. Signal Transduct. Target. Ther..

[16] Warnatsch A (2017). Reactive oxygen species localization programs inflammation to clear microbes of different size. Immunity.

[17] Kolaczkowska E, Kubes P (2013). Neutrophil recruitment and function in health and inflammation. Nat. Rev. Immunol..

[18] Urban CF, Reichard U, Brinkmann V, Zychlinsky A (2006). Neutrophil extracellular traps capture and kill Candida albicans yeast and hyphal forms. Cell. Microbiol..

[19] Romani L (2004). Immunity to fungal infections. Nat. Rev. Immunol..

[20] Schimke LF (2017). Paracoccidioidomycosis Associated with a Heterozygous STAT4 Mutation and Impaired IFN-γ Immunity. J. Infect. Dis..

[21] Seurat - Guided Clustering Tutorial. https://satijalab.org/seurat/articles/pbmc3k_tutorial.html (2021).

[22] Chaussabel D, Baldwin N (2014). Democratizing systems immunology with modular transcriptional repertoire analyses. Nat. Rev. Immunol..

[23] WGCNA package: Frequently Asked Questions. https://horvath.genetics.ucla.edu/html/CoexpressionNetwork/Rpackages/WGCNA/faq.html.

[24] Russo PST (2018). CEMiTool: A Bioconductor package for performing comprehensive modular coexpression analyses. BMC Bioinform..

[25] Yu G, Wang LG, Han Y, He QY (2012). ClusterProfiler: An R package for comparing biological themes among gene clusters. Omi. A J. Integr. Biol..

[26] Bruno M (2021). Data of common and species-specific transcriptional host responses to pathogenic fungi. Data Br..

[27] Jaeger M (2015). The RIG-I-like helicase receptor MDA5 (IFIH1) is involved in the host defense against Candida infections. Eur. J. Clin. Microbiol. Infect. Dis..

[28] Bruno M (2020). Transcriptional and functional insights into the host immune response against the emerging fungal pathogen Candida auris. Nat. Microbiol..

[29] Dix A (2015). Biomarker-based classification of bacterial and fungal whole-blood infections in a genome-wide expression study. Front. Microbiol..

[30] Sieber P (2018). Comparative study on alternative splicing in human fungal pathogens suggests its involvement during host invasion. Front. Microbiol..

[31] Kämmer, P. *et al.* Survival strategies of pathogenic Candida species in human blood show independent and specific adaptations. *MBio* **11**, (2020). 10.1128/mBio.02435-20 PMC7542370 33024045

[32] Ramesh N, Salama M, Dangott B, Tasdizen T (2012). Isolation and two-step classification of normal white blood cells in peripheral blood smears. J. Pathol. Inform..

[33] De Vito R, Bellio R, Trippa L, Parmigiani G (2019). Multi-study factor analysis. Biometrics.

[34] Dix A (2017). Specific and novel microRNAs are regulated as response to fungal infection in human dendritic cells. Front. Microbiol..

[35] Rizzetto L (2010). Differential IL-17 production and mannan recognition contribute to fungal pathogenicity and commensalism. J. Immunol..

[36] Rizzetto L (2012). The modular nature of dendritic cell responses to commensal and pathogenic fungi. PLoS ONE.

[37] Bourgeois C (2011). Conventional Dendritic Cells Mount a Type I IFN Response against Candida spp. Requiring Novel Phagosomal TLR7-Mediated IFN-β Signaling. J. Immunol..

[38] Romani L, Puccetti P (2007). Controlling pathogenic inflammation to fungi. Expert Rev. Anti. Infect. Ther..

[39] Romani L, Zelante T, De Luca A, Fallarino F, Puccetti P (2008). IL-17 and therapeutic kynurenines in pathogenic inflammation to fungi. J. Immunol..

[40] Aggarwal R, Ranganathan P (2016). Common pitfalls in statistical analysis: The use of correlation techniques. Perspect. Clin. Res..

[41] Rickman JM, Wang Y, Rollett AD, Harmer MP, Compson C (2017). Data analytics using canonical correlation analysis and Monte Carlo simulation. NPJ Comput. Mater..

[42] Arendrup MC, Patterson TF (2017). Multidrug-resistant Candida: Epidemiology, molecular mechanisms, and treatment. J. Infect. Dis..

[43] Tangye SG (2020). Human inborn errors of immunity: 2019 Update on the classification from the international union of immunological societies expert committee. J. Clin. Immunol..

[44] Vallabhajosyula RR, Chakravarti D, Lutfeali S, Ray A, Raval A (2009). Identifying hubs in protein interaction networks. PLoS ONE.

[45] Kawai T, Akira S (2007). Signaling to NF-κB by Toll-like receptors. Trends Mol. Med..

[46] Tsai, M.-H., Pai, L.-M. & Lee, C.-K. Fine-Tuning of type I interferon response by STAT3. *Front. Immunol.* **10**, (2019). 10.3389/fimmu.2019.01448 PMC6606715 31293595

[47] Balic JJ (2020). STAT3 serine phosphorylation is required for TLR4 metabolic reprogramming and IL-1β expression. Nat. Commun..

[48] Luu K (2014). STAT1 plays a role in TLR signal transduction and inflammatory responses. Immunol. Cell Biol..

[49] Ramana CV, Gil MP, Schreiber RD, Stark GR (2002). Stat1-dependent and -independent pathways in IFN-γ-dependent signaling. Trends Immunol..

[50] Rhee SH, Jones BW, Toshchakov V, Vogel SN, Fenton MJ (2003). Toll-like receptors 2 and 4 activate STAT1 serine phosphorylation by distinct mechanisms in macrophages. J. Biol. Chem..

[51] Hardison SE, Brown GD (2012). C-type lectin receptors orchestrate antifungal immunity. Nat. Immunol..

[52] Muñoz JF (2019). Coordinated host-pathogen transcriptional dynamics revealed using sorted subpopulations and single macrophages infected with Candida albicans. Nat. Commun..

[53] Qiao Y (2013). Synergistic activation of inflammatory cytokine genes by interferon-γ-induced chromatin remodeling and toll-like receptor signaling. Immunity.

[54] Donini M, Zenaro E, Tamassia N, Dusi S (2007). NADPH oxidase of human dendritic cells: Role inCandida albicans killing and regulation by interferons, dectin-1 and CD206. Eur. J. Immunol..

[55] Riedelberger M (2020). Type I interferons ameliorate zinc intoxication of Candida glabrata by macrophages and promote fungal immune evasion. iScience.

[56] Riedelberger M (2020). Type I interferon response dysregulates host iron homeostasis and enhances Candida glabrata infection. Cell Host Microbe.

[57] Patin EC (2016). IL-27 induced by select candida spp. via TLR7/NOD2 signaling and IFN-β production inhibits fungal clearance. J. Immunol..

[58] Cardone M (2014). Interleukin-1 and Interferon-γ Orchestrate β-Glucan-activated human dendritic cell programming via IκB-ζ modulation. PLoS ONE.

[59] del Fresno C (2013). Interferon-β Production via Dectin-1-Syk-IRF5 Signaling in Dendritic Cells Is Crucial for Immunity to C. albicans. Immunity.

[60] Biondo C (2011). Recognition of yeast nucleic acids triggers a host-protective type I interferon response. Eur. J. Immunol..

[61] Li, T., Niu, X., Zhang, X., Wang, S. & Liu, Z. Recombinant human IFNα-2b response promotes vaginal epithelial cells defense against Candida albicans. *Front. Microbiol.* **8**, (2017). 10.3389/fmicb.2017.00697 PMC5397410 28473823

[62] Li T, Liu Z, Zhang X, Chen X, Wang S (2019). Therapeutic effectiveness of type I interferon in vulvovaginal candidiasis. Microb. Pathog..

[63] Pekmezovic M (2021). Candida pathogens induce protective mitochondria-associated type I interferon signaling and a damage-driven response in vaginal epithelial cells. Nat. Microbiol..

[64] Majer O (2012). Type I interferons promote fatal immunopathology by regulating inflammatory monocytes and neutrophils during Candida infections. PLoS Pathog..

[65] Break TJ (2021). Aberrant type 1 immunity drives susceptibility to mucosal fungal infections. Science.

[66] Okada S (2020). Human STAT1 gain-of-function heterozygous mutations: Chronic mucocutaneous candidiasis and type I interferonopathy. J. Clin. Immunol..

[67] Stawowczyk, M. *et al.* Pathogenic effects of IFIT2 and interferon-β during fatal systemic Candida albicans infection. *MBiol* **9**, (2018). 10.1128/mBio.00365-18 PMC5904408 29666281

[68] Hu X, Chen J, Wang L, Ivashkiv LB (2007). Crosstalk among Jak-STAT, Toll-like receptor, and ITAM-dependent pathways in macrophage activation. J. Leukoc. Biol..

[69] Bohnenkamp HR, Papazisis KT, Burchell JM, Taylor-Papadimitriou J (2007). Synergism of Toll-like receptor-induced interleukin-12p70 secretion by monocyte-derived dendritic cells is mediated through p38 MAPK and lowers the threshold of T-helper cell type I responses. Cell. Immunol..

[70] Makela SM, Strengell M, Pietila TE, Osterlund P, Julkunen I (2009). Multiple signaling pathways contribute to synergistic TLR ligand-dependent cytokine gene expression in human monocyte-derived macrophages and dendritic cells. J. Leukoc. Biol..

[71] Tong Y (2012). Enhanced TLR-induced NF-κB signaling and type I interferon responses in NLRC5 deficient mice. Cell Res..

[72] Bosisio D (2002). Stimulation of toll-like receptor 4 expression in human mononuclear phagocytes by interferon-γ: A molecular basis for priming and synergism with bacterial lipopolysaccharide. Blood.

[73] Schroder K (2007). PU1 and ICSBP control constitutive and IFN-γ-regulated Tlr9 gene expression in mouse macrophages. J. Leukoc. Biol..

[74] Kajita A, i. (2015). Interferon-gamma enhances TLR3 expression and anti-viral activity in keratinocytes. J. Invest. Dermatol..

[75] Mita Y (2002). Toll-like receptor 4 surface expression on human monocytes and B cells is modulated by IL-2 and IL-4. Immunol. Lett..

[76] Rodrigues ML, Nosanchuk JD (2020). Fungal diseases as neglected pathogens: A wake-up call to public health officials. PLoS Negl. Trop. Dis..

[77] Casanova J-L, Abel L (2021). Lethal infectious diseases as inborn errors of immunity: toward a synthesis of the germ and genetic theories. Annu. Rev. Pathol. Mech. Dis..

[78] Notarangelo LD, Bacchetta R, Casanova J-L, Su HC (2020). Human inborn errors of immunity: An expanding universe. Sci. Immunol..

[79] Casanova J-L, Abel L (2004). The human model: A genetic dissection of immunity to infection in natural conditions. Nat. Rev. Immunol..

[80] Joly S (2009). Cutting edge: Candida albicans hyphae formation triggers activation of the Nlrp3 inflammasome. J. Immunol..

[81] Schönherr FA (2017). The intraspecies diversity of C. albicans triggers qualitatively and temporally distinct host responses that determine the balance between commensalism and pathogenicity. Mucosal Immunol..

[82] Edgar R, Domrachev M, Lash AE (2002). Gene expression omnibus: NCBI gene expression and hybridization array data repository. Nucleic Acids Res..

[83] Athar A (2019). ArrayExpress update—from bulk to single-cell expression data. Nucleic Acids Res..

[84] de Vries DH (2020). Integrating GWAS with bulk and single-cell RNA-sequencing reveals a role for LY86 in the anti-Candida host response. PLOS Pathog..

[85] Stuart T (2019). Comprehensive integration of single-cell data. Cell.

[86] Zhou G (2019). NetworkAnalyst 30: A visual analytics platform for comprehensive gene expression profiling and meta-analysis. Nucleic Acids Res..

[87] Barrett T (2012). NCBI GEO: Archive for functional genomics datasets—update. Nucleic Acids Res..

[88] Law CW, Chen Y, Shi W, Smyth GK (2014). voom: Precision weights unlock linear model analysis tools for RNA-seq read counts. Genome Biol..

[89] Kuleshov MV (2016). Enrichr: A comprehensive gene set enrichment analysis web server 2016 update. Nucleic Acids Res..

[90] Khan A, Mathelier A (2017). Intervene: A tool for intersection and visualization of multiple gene or genomic region sets. BMC Bioinform..

[91] Starruß J, de Back W, Brusch L, Deutsch A (2014). Morpheus: A user-friendly modeling environment for multiscale and multicellular systems biology. Bioinformatics.

[92] Walter W, Sánchez-Cabo F, Ricote M (2015). GOplot: An R package for visually combining expression data with functional analysis: Fig. 1. Bioinformatics.

[93] Wickham, H. Getting Started with ggplot2. in 11–31 (2016). 10.1007/978-3-319-24277-4_2.

[94] Gu Z, Eils R, Schlesner M (2016). Complex heatmaps reveal patterns and correlations in multidimensional genomic data. Bioinformatics.

[95] Jendoubi T, Strimmer K (2019). A whitening approach to probabilistic canonical correlation analysis for omics data integration. BMC Bioinformatics.

[96] Brown KR (2009). NAViGaTOR: Network analysis, visualization and graphing Toronto. Bioinformatics.

[97] Kotlyar M, Pastrello C, Malik Z, Jurisica I (2019). IID 2018 update: Context-specific physical protein–protein interactions in human, model organisms and domesticated species. Nucleic Acids Res..

[98] Pastrello, C., Kotlyar, M. & Jurisica, I. Informed use of protein–protein interaction data: A focus on the integrated interactions database (IID). 125–134 (2020). 10.1007/978-1-4939-9873-9_10. 10.1007/978-1-4939-9873-9_10 31583635

[99] De Vito, R., Bellio, R., Trippa, L. & Parmigiani, G. Bayesian Multistudy Factor Analysis for High-throughput Biological Data. (2018).

[100] Cabral-Marques O (2018). GPCR-specific autoantibody signatures are associated with physiological and pathological immune homeostasis. Nat. Commun..

[101] Dempster AP, Laird NM, Rubin DB (1977). Maximum likelihood from incomplete data via the EM algorithm. J. R. Stat. Soc. Ser. B.

