# Supplementary material for: The network interplay of interferon and Toll-like receptor signaling pathways in the anti-Candida immune response
Source: Sci Rep. 2021 Oct 13;11:20281. doi: 10.1038/s41598-021-99838-0 (PMC8514550; doi:10.1038/s41598-021-99838-0)
Supplement: Supplementary file 1 — Supplementary Information 1. [file 41598_2021_99838_MOESM1_ESM.pdf]

Suppl. Fig. 1

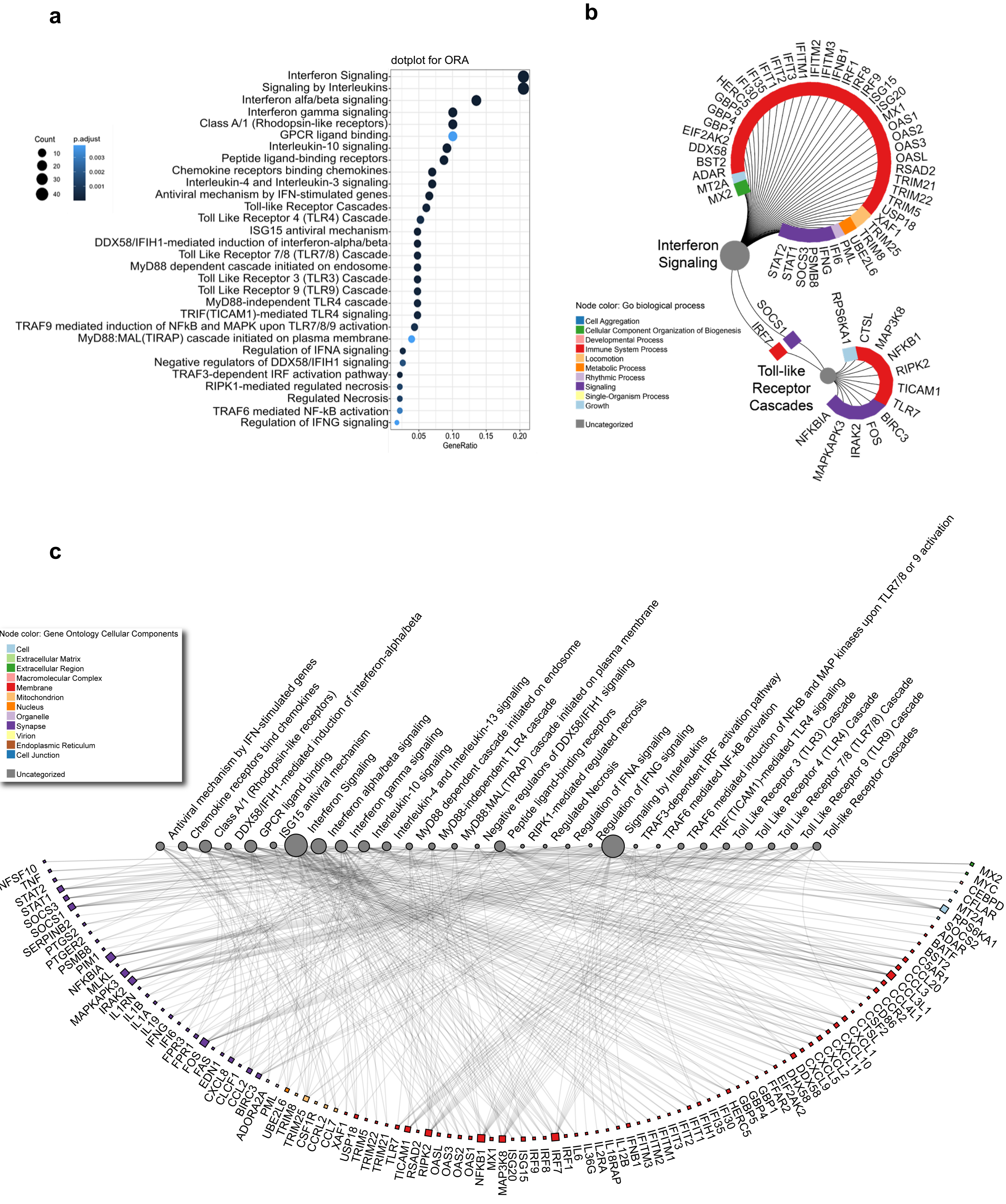

a
dotplot for ORA
Interferon Signaling
Signaling by Interleukins
Interferon alfa/beta signaling
Interferon gamma signaling
Class A/1 (Rhodopsin-like receptors)
GPCR ligand binding
Interleukin-10 signaling
Peptide ligand-binding receptors
Chemokine receptors binding chemokines
Interleukin-4 and Interleukin-3 signaling
Antiviral mechanism by IFN-stimulated genes
Toll-like Receptor Cascades
Toll Like Receptor 4 (TLR4) Cascade
ISG15 antiviral mechanism
DDX58/IFIH1-mediated induction of interferon-alpha/beta
Toll Like Receptor 7/8 (TLR7/8) Cascade
MyD88 dependent cascade initiated on endosome
Toll Like Receptor 3 (TLR3) Cascade
Toll Like Receptor 9 (TLR9) Cascade
MyD88-independent TLR4 cascade
TRIF(TICAM1)-mediated TLR4 signaling
TRAF9 mediated induction of NFkB and MAPK upon TLR7/8/9 activation
MyD88:MAL(TIRAP) cascade initiated on plasma membrane
Regulation of IFNA signaling
Negative regulators of DDX58/IFIH1 signaling
TRAF3-dependent IRF activation pathway
RIPK1-mediated regulated necrosis
Regulated Necrosis
TRAF6 mediated NF-kB activation
Regulation of IFNG signaling
0.05
0.10
0.15
0.20
GeneRatio
Count
10
20
30
40
p.adjust
0.003
0.002
0.001
b
Interferon Signaling
Toll-like Receptor Cascades
Node color: Go biological process
Cell Aggregation
Cellular Component Organization of Biogenesis
Developmental Process
Immune System Process
Locomotion
Metabolic Process
Rhythmic Process
Signaling
Single-Organism Process
Growth
Uncategorized
HERC5 GBP5 GBP4 GBP1 EIF2AK2 DDX58 BST2 ADAR MT2A MX2 IFI30 IFI35 IFIT1 IFIT2 IFIT3 IFITM1 IFITM2 IFITM3 IFNB1 IRF1 IRF8 IRF9 ISG15 ISG20 MX1 OAS1 OAS2 OAS3 OASL RSAD2 TRIM21 TRIM22 TRIM5 USP18 XAF1 TRIM25 TRIM8 UBE2L6 PML IFI6 IFNG PSMB8 SOCS3 STAT1 STAT2
SOCS1 IRF7 RPS6KA1 CTSL MAP3K8 NFKB1 RIPK2 TICAM1 TLR7 BIRC3 FOS IRAK2 MAPKAPK3 NFKBIA
c
Node color: Gene Ontology Cellular Components
Cell
Extracellular Matrix
Extracellular Region
Macromolecular Complex
Membrane
Mitochondrion
Nucleus
Organelle
Synapse
Virion
Endoplasmic Reticulum
Cell Junction
Uncategorized
Antiviral mechanism by IFN-stimulated genes
Chemokine receptors bind chemokines
Class A/1 (Rhodopsin-like receptors)
DDX58/IFIH1-mediated induction of interferon-alpha/beta
GPCR ligand binding
ISG15 antiviral mechanism
Interferon Signaling
Interferon alpha/beta signaling
Interferon gamma signaling
Interleukin-10 signaling
Interleukin-4 and Interleukin-13 signaling
MyD88 dependent cascade initiated on endosome
MyD88-independent TLR4 cascade
MyD88:MAL(TIRAP) cascade initiated on plasma membrane
Negative regulators of DDX58/IFIH1 signaling
Peptide ligand-binding receptors
RIPK1-mediated regulated necrosis
Regulated Necrosis
Regulation of IFNA signaling
Regulation of IFNG signaling
Signaling by Interleukins
TRAF3-dependent IRF activation pathway
TRAF6 mediated NF-kB activation
TRAF6 mediated induction of NFkB and MAP kinases upon TLR7/8 or 9 activation
TRIF(TICAM1)-mediated TLR4 signaling
Toll Like Receptor 3 (TLR3) Cascade
Toll Like Receptor 4 (TLR4) Cascade
Toll Like Receptor 7/8 (TLR7/8) Cascade
Toll Like Receptor 9 (TLR9) Cascade
Toll-like Receptor Cascades
TNFSF10 TNF STAT2 STAT1 SOCS3 SOCS1 SERPINB2 PTGS2 PTGER2 PSMB8 PIM1 NFKBIA MLKL MAPKAPK3 IRAK2 IL1RN IL1B IL1A IL19 IFNG IFI6 FPR3 FPR1 FOS FAS EDN1 CXCL8 CLCF1 CCL2 BIRC3 ADORA2A PML UBE2L6 TRIM8 TRIM25 CSF1R CCRL2 CCL7 XAF1 USP18 TRIM5 TRIM22 TRIM21 TLR7 TICAM1 RSAD2 RIPK2 OASL OAS3 OAS2 OAS1 NFKB1 MX1 MAP3K8 ISG20 ISG15 IRF9 IRF8 IRF7 IRF1 IL6 IL36G IL2RA IL18RAP IL12B IFNB1 IFITM3 IFITM2 IFITM1 IFIT3 IFIT2 IFIT1 IFIH1 IFI35 IFI30 HERC5 GBP5 GBP4 GBP1 FFAR2 EIF2AK2 DHX58 DDX58 CXCL9 CXCL5 CXCL2 CXCL11 CXCL10
MX2 MYC CEBPD TLR2 MT2A RPS6KA1 SOCS2 ADAR BATF BST2

Suppl. Fig. 1. Functional clustering of DEGs associated with TLR and IFN signaling pathways. a, Dot plot showing the 30 most enriched signaling pathways obtained by ORA of DEGs (dataset GSE42606) using ClusterProfiler. The Y-axis contains enriched pathways; the size of circles represents the number of genes (count) enriching each category, and the color (from blue to black) indicates how significantly (when p value < 0.05) enriched the pathway is. **b,** Network of TLR- and IFN-associated gene enrichment signaling pathways shown in the dot plot (a). The network includes upregulated and downregulated genes (**Suppl. Table S4**) comparing resting with *C. albicans*-infected PBMCs. The size of circles represents the number (counts) of genes enriched in the pathway, and colored squares represent the cell location of genes. **c,** Network demonstrating interactions between pathways and their associated genes revealed by ORA. Circular nodes represent pathways; the circle size is associated with the number of genes that enrich each pathway, and colored squares represent the cell location of genes. The interaction network was built using NAViGaTOR software. *DEGs, differentially expressed genes; IFN, interferon; ORA, overrepresentation analysis; TLR, Toll-like receptor.*

Suppl. Fig. 2

a

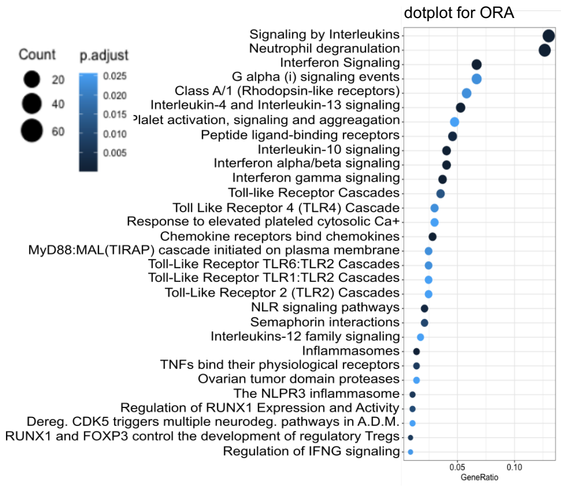

b

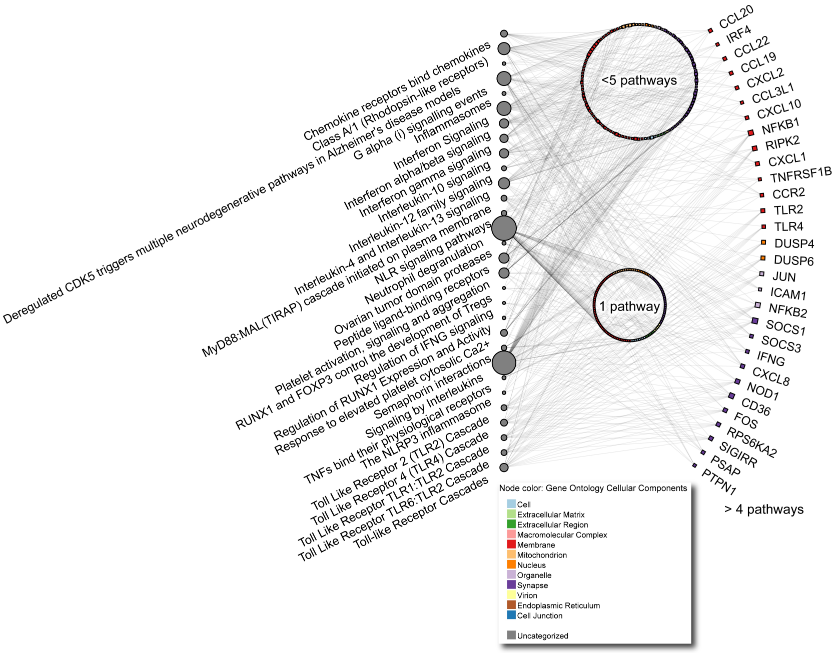

Suppl. Fig. 2. Induction of TLR and IFN signaling pathways by *Candida auris*. a, Dot plot showing the 30 most enriched signaling pathways obtained by ORA of DEGs using ClusterProfiler. The Y-axis contains enriched pathways; the size of circles represents the number of genes (count) enriched in each category, and the color (from blue to black) indicates how significantly enriched (when p-value <0.05) the pathway is. **b,** Network demonstrating interactions between pathways and their associated genes revealed by ORA. Circular nodes represent pathways, circle size is associated with the number of genes enriching each pathway, and colored squares represent the cell location of genes. Genes interacting with more than 5 pathways are indicated. The interaction network was built using NAViGaTOR software.

Suppl. Fig. 3

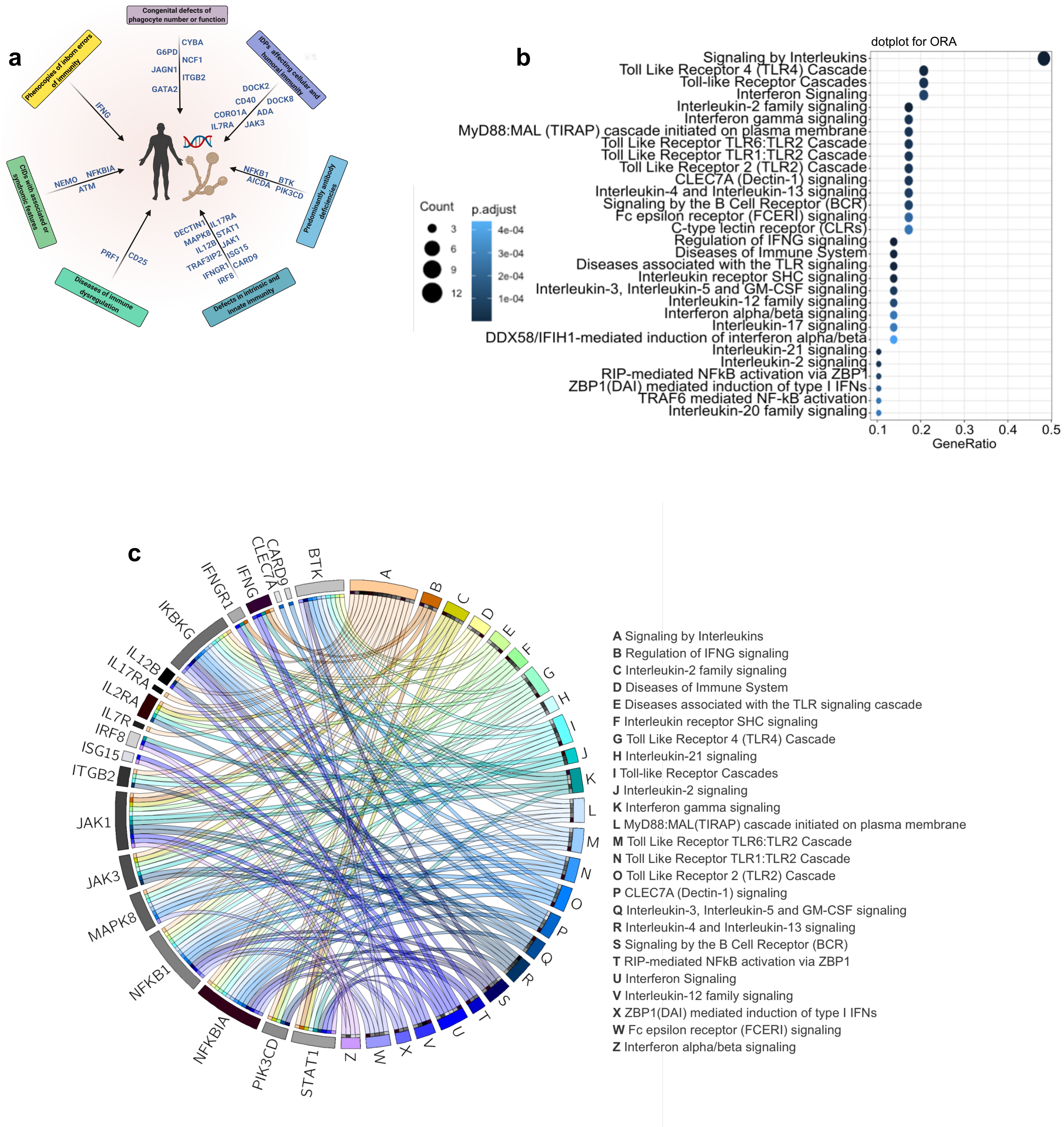
a
Congenital defects of phagocyte number or function
CYBA
G6PD
NCF1
JAGN1
ITGB2
GATA2
IDPs affecting cellular and humoral immunity
DOCK2
CD40
DOCK8
CORO1A
ADA
IL7RA
JAK3
Phenocopies of inborn errors of immunity
IFNG
CIDs with associated or syndromic features
NEMO
NFKBIA
ATM
Predominantly antibody deficiencies
NFKB1
BTK
AICDA
PIK3CD
Diseases of immune dysregulation
PRF1
CD25
Defects in intrinsic and innate immunity
DECTIN1
IL17RA
MAPK8
STAT1
IL12B
JAK1
TRAF3IP2
ISG15
IFNGR1
CARD9
IRF8
b
dotplot for ORA
Signaling by Interleukins
Toll Like Receptor 4 (TLR4) Cascade
Toll-like Receptor Cascades
Interferon Signaling
Interleukin-2 family signaling
Interferon gamma signaling
MyD88:MAL (TIRAP) cascade initiated on plasma membrane
Toll Like Receptor TLR6:TLR2 Cascade
Toll Like Receptor TLR1:TLR2 Cascade
Toll Like Receptor 2 (TLR2) Cascade
CLEC7A (Dectin-1) signaling
Interleukin-4 and Interleukin-13 signaling
Signaling by the B Cell Receptor (BCR)
Fc epsilon receptor (FCERI) signaling
C-type lectin receptor (CLRs)
Regulation of IFNG signaling
Diseases of Immune System
Diseases associated with the TLR signaling
Interleukin receptor SHC signaling
Interleukin-3, Interleukin-5 and GM-CSF signaling
Interleukin-12 family signaling
Interferon alpha/beta signaling
Interleukin-17 signaling
DDX58/IFIH1-mediated induction of interferon alpha/beta
Interleukin-21 signaling
Interleukin-2 signaling
RIP-mediated NFkB activation via ZBP1
ZBP1(DAI) mediated induction of type I IFNs
TRAF6 mediated NF-kB activation
Interleukin-20 family signaling
Count
3
6
9
12
p.adjust
4e-04
3e-04
2e-04
1e-04
0.1
0.2
0.3
0.4
0.5
GeneRatio
c
BTK
CARD9
CLEC7A
IFNG
IFNGR1
IKBKG
IL12B
IL17RA
IL2RA
IL7R
IRF8
ISG15
ITGB2
JAK1
JAK3
MAPK8
NFKB1
NFKBIA
PIK3CD
STAT1
A B C D E F G H I J K L M N O P Q R S T U V X W Z
A Signaling by Interleukins
B Regulation of IFNG signaling
C Interleukin-2 family signaling
D Diseases of Immune System
E Diseases associated with the TLR signaling cascade
F Interleukin receptor SHC signaling
G Toll Like Receptor 4 (TLR4) Cascade
H Interleukin-21 signaling
I Toll-like Receptor Cascades
J Interleukin-2 signaling
K Interferon gamma signaling
L MyD88:MAL(TIRAP) cascade initiated on plasma membrane
M Toll Like Receptor TLR6:TLR2 Cascade
N Toll Like Receptor TLR1:TLR2 Cascade
O Toll Like Receptor 2 (TLR2) Cascade
P CLEC7A (Dectin-1) signaling
Q Interleukin-3, Interleukin-5 and GM-CSF signaling
R Interleukin-4 and Interleukin-13 signaling
S Signaling by the B Cell Receptor (BCR)
T RIP-mediated NFkB activation via ZBP1
U Interferon Signaling
V Interleukin-12 family signaling
X ZBP1(DAI) mediated induction of type I IFNs
W Fc epsilon receptor (FCERI) signaling
Z Interferon alpha/beta signaling

Suppl. Fig. 3. Inborn errors of immunity confirm interplay of TLR- and IFN-associated genes. a, Overview (created using BioRender.com) of genes associated with inborn errors of immunity that were also found among the DEGs across the 7 datasets of WBCs, PBMCs, and moDCs (**Suppl. Table S16**). **b,** Dot plot showing the 30 most enriched signaling pathways obtained by ORA of the DEGs shown in (**a**). The Y-axis contains enriched pathways; the size of circles represents the number of genes (count) enriched in each category, and the color (from blue to black) indicates how significantly enriched (when p-value <0.05) each pathway is. **c,** GOPlot displaying genes causing inborn errors of immunity and enriched pathways. *DEGs, differentially expressed genes; moDCs, monocyte-derived dendritic cells; IFN, interferon; ORA, overrepresentation analysis; PBMCs, peripheral blood mononuclear cells; TLR, Toll-like receptor; WBCs, whole blood cells.*
